# Non-Clinical Evaluation of the Anti-PD-1 Antibody UDIZ-007

**DOI:** 10.3390/ph19091423

**Published:** 2026-09-09

**Authors:** Gregorio de Jesús Carballo Uicab, Keyla María Gómez Castellano, Frida Daniela Ramírez Villedas, Marco A. Velasco Velázquez, Ileana Licona-Limón, Said Kayum Vázquez Leyva, Hugo Alberto Barrera Saldaña, Sonia Mayra Pérez Tapia, Juan Carlos Almagro

**Affiliations:** 1 Unidad de Desarrollo e Investigación en Bioterapéuticos (UDIBI), Escuela Nacional de Ciencias Biológicas, Instituto Politécnico Nacional, Mexico City 11340, Mexico; gregorio.carballo@udibi.com.mx (G.d.J.C.U.); keyla.gomez@udibi.com.mx (K.M.G.C.); frida.ramirez@udibi.com.mx (F.D.R.V.); ileana.licona@udibi.com.mx (I.L.-L.); said.vazquez@udibi.com.mx (S.K.V.L.); 2 Laboratorio Nacional Para Servicios Especializados de Investigación, Desarrollo e Innovación (I+D+I) Para Farmoquímicos y Biotecnológicos, LANSEIDI-FarBiotec-CONACyT, Mexico City 11340, Mexico; habarrera@gmail.com; 3 Facultad de Medicina, Universidad Nacional Autónoma de México, Mexico City 04510, Mexico; marcovelasco@unam.mx; 4 Dirección de Investigación Científica Básica, Laboratorios Columbia Comercial SA de CV, Mexico City 04000, Mexico; 5 Departamento de Inmunología, Escuela Nacional de Ciencias Biológicas, Instituto Politécnico Nacional (ENCB-IPN), Mexico City 11350, Mexico; 6 GlobalBio, Inc., Cambridge, MA 02138, USA

**Keywords:** PD-1, anti-tumor activity, pharmacokinetics, immunogenicity, cancer immunotherapy

## Abstract

**Background/Objectives**: Programmed cell death protein 1 (PD-1) is a key immune checkpoint that suppresses anti-tumor T-cell responses and is an established target for cancer immunotherapy. UDIZ-007 is a fully human anti-PD-1 antibody that blocks PD-1/PD-L1 and PD-1/PD-L2 interactions and eradicates MC38-hPD-L1 colon tumors in B-hPD-1 transgenic mice. This study further characterized the pharmacokinetics (PK), immunogenicity, and safety of UDIZ-007 to support its clinical development. **Methods**: UDIZ-007 was produced in a stable Chinese hamster ovary (CHO) cell line, and its biophysical and in vitro functional profiles were assessed. PK, immunogenicity, and safety were evaluated in mice and cynomolgus macaques. **Results**: UDIZ-007 showed biophysical and in vitro functional properties consistent with therapeutic antibodies and demonstrated potent, dose-dependent anti-tumor activity against MC38-hPD-L1 colon tumors engrafted in B-hPD-1 transgenic mice. Single-dose PK studies in mice at 10 and 100 mg/kg and in cynomolgus macaques at 10, 50, and 101.2 mg/kg showed a trend toward dose-proportional exposure. In cynomolgus macaques, UDIZ-007 had a prolonged half-life of approximately 12 days. Repeated intravenous administration at 10, 50, or 101.2 mg/kg in cynomolgus macaques was generally well tolerated, with no major treatment-related toxicities. Accordingly, the no-observed-adverse-effect level (NOAEL) was established at the highest tested dose, 101.2 mg/kg. Anti-drug antibodies (ADAs) were detected in some animals and were associated with expected PK variability but not with adverse effects. Tissue cross-reactivity studies across 32 human and cynomolgus macaque tissues showed binding primarily restricted to lymphoid tissues known to express PD-1. **Conclusions**: UDIZ-007 demonstrated robust anti-tumor activity, favorable PK and safety profiles, as well as selective binding to PD-1-expressing tissues, supporting its clinical development as a potential cancer immunotherapy.

## 1. Introduction

Globally, cancer is a major and increasing public health burden, with approximately 20 million new cases and 9.7 million cancer-related deaths estimated in 2022 [1]. This burden is expected to rise substantially over the coming decades due to population growth and aging, with annual new cases projected to reach about 35 million by 2050. This trend is particularly relevant for regions such as Latin America and the Caribbean [2], where rising incidence, substantial mortality, and persistent disparities in access to timely diagnosis and effective treatment continue to affect outcomes. The expanding global and regional cancer burdens have, therefore, underscored the need for continued development of therapeutic strategies that can broaden clinical benefit, improve treatment options across tumor types, and support more equitable access to effective cancer therapies.

The seminal work of Tasuku Honjo in the 1990s demonstrated that blocking the interaction between PD-1 (Programmed cell death protein 1, CD279), with its ligands PD-L1 and PD-L2 (Programmed cell death ligands 1 and 2; CD274 and CD273, respectively), could elicit anti-tumor responses, highlighting the therapeutic potential of PD-1/PD-L1 blockade across multiple malignancies [3,4]. This groundbreaking work led to the development of the anti-PD-1 blockbuster drugs pembrolizumab and nivolumab, both approved by the US Food and Drug Administration (FDA) in 2014 for the treatment of cancer [5,6]. The clinical success of pembrolizumab and nivolumab spurred an explosion in the discovery, engineering, and development of new anti-PD-1 therapeutic antibodies over the past decade. Currently, 16 anti-PD-1-based monotherapies have been approved globally for cancer treatment in diverse malignancies [7]. However, none of these antibodies have been developed in Latin America and the Caribbean, limiting regional access to these therapeutic options.

The approved therapeutic anti-PD-1 antibodies have been developed using different engineering methods, have been characterized by diverse biochemical and cell-based assays, and have followed distinct regulatory pathways [7]. For instance, 10 of the 16 monospecific antibodies (63%) have humanized variable (V) regions. The remaining six are fully human antibodies obtained from transgenic mice or rats or have been selected via phage or mammalian display technologies. Human IgG4 has been the isotype of choice for engineering the vast majority (14 out of 16; 87%) of the anti-PD-1 antibodies, with only two antibodies, prolgolimab and penpulimab, having IgG1 isotypes engineered with LALA mutations [7]. The IgG1 isotype may confer higher stability and higher production yields, while the LALA mutations abrogate effector functions [8].

From a functional viewpoint, although all clinically approved anti-PD-1 antibodies bind the front face and the membrane distal region of human PD-1 (hPD-1), blocking the interaction with PD-L1 and PD-L2, the fine details of their interaction with hPD-1 indicate diverse mechanisms of binding [7]. For instance, the flexibility of the N-terminal region of hPD-1 plays an important role in the interaction with nivolumab [9]. The glycan at position N58 of hPD-1 affects binding with other antibodies [10,11]. Further, an anti-PD-1 antibody, called NBO1a [12], that has not yet been approved for clinical use, recognizes an epitope that does not overlap with the PD-L1 binding site nor with the epitope recognized by pembrolizumab. The combination of NBO1a with pembrolizumab has been reported to enhance anti-tumor activity over pembrolizumab in specific experimental settings [12]. On the other hand, one of the therapeutic antibodies, serplulimab, has shown enhanced tumor killing activity upon co-administration with anti-TIGIT or anti-LAG3 inhibitors [13]. Therefore, combinations of anti-PD-1 antibodies with distinct mechanisms of binding and/or targeting diverse oncology relevant molecules are expected to broaden clinical benefit and/or expand the therapeutic options of currently approved antibodies for clinical use to meet the growing demand for cancer treatment.

In a previous report [14], we described the isolation and initial characterization of a novel anti-PD-1 antibody, called UDIZ-007. The V regions of UDIZ-007 were isolated from a human antibody phage display library and reformatted as human IgG4 PE and IgG1 LALA, with the latter improving yield and enhancing solubility in formulation buffers. UDIZ-007 effectively blocked the PD-1/PD-L1 and PD-1/PD-L2 interactions [14] and showed high selectivity since it does not cross-react with CD28 receptor family members. In preliminary efficacy studies, six intraperitoneal (IP) administrations of UDIZ-007 (10 mg/kg every three days) eradicated MC38-hPD-L1 colon tumors engrafted in B-hPD-1 transgenic mice by day 17, with no tumor relapse at the end point (day 56). Importantly, UDIZ-007 recognized a unique region of hPD-1 [14] with low overlap with the PD-L1/L2 binding sites, leading to the hypothesis that it may broaden current therapeutic options such as monotherapy or enhance efficacy in combination with already clinically approved anti-PD-1 therapeutic antibodies.

With the aim of testing the clinical efficacy of UDIZ-007 in future studies, in this report, we conducted the preclinical development of this promising therapeutic antibodyproduced in a stable CHO cell line as a non-GMP drug substance preparation (UDIZ-007 DS-nGMP). We first evaluated its biophysical profile and functionality in vitro. Second, we expanded our previous in vivo efficacy study by evaluating the anti-tumor activity of UDIZ-007 DS-nGMP in the MC38-hPD-L1 tumor model in B-hPD-1 transgenic mice at three dose levels, 1, 2.5, and 10 mg/kg, administered intraperitoneally (IP). Third, pharmacokinetic (PK) analyses were conducted in both B-hPD-1 transgenic mice and cynomolgus macaques. The non-human primate studies included single intravenous (IV) administrations of 10, 50, and 101.2 mg/kg, as well as repeated weekly dosing at the same dose levels via IV administration to evaluate safety and tolerability. Fourth, anti-drug antibody (ADA) responses were evaluated in cynomolgus macaques following both single and repeated antibody administrations. Finally, cross-reactivity studies in human and cynomolgus macaque tissues (TCR) were performed to further assess the translational safety profile of UDIZ-007. We concluded that UDIZ-007 DS-nGMP exhibits potent dose-dependent anti-tumor activity together with favorable pharmacological and safety profiles, thus supporting the advancement of UDIZ-007 toward clinical evaluation.

## 2. Results

### 2.1. Biophysical Characterization of UDIZ-007 DS-nGMP

SDS-PAGE, SEC-UPLC and intact MS analysis are provided in the Supplementary Materials, Figure S1. The SDS-PAGE analysis under non-reducing conditions showed a predominant band corresponding to an intact IgG with an apparent molecular weight of approximately 140.8 kDa. Under reducing conditions, two major bands were observed at approximately 47.1 and 25.1 kDa corresponding to the heavy and light chains, respectively. SEC-UPLC analysis demonstrated a highly homogeneous preparation with a predominant monomeric peak corresponding to an apparent molecular weight of 176.21 kDa and an estimated monomeric content of approximately 100%. Intact mass analysis identified G0F/G0F as the predominant glycoform with 146,855 Da, alongside additional minor glycoforms corresponding to G0F/G1F, G0/G1F, G1F/G1F species with 147,019, 146,648 and 147,182 Da, respectively. Finally, peptide mapping analyses confirmed the primary amino acid sequence of UDIZ-007 and its disulfide bond connectivity.

The in vitro functional characterization of UDIZ-007 DS-nGMP was evaluated by SPR, BLI, and hPD-1/PD-L1 blockade in a cell-based assay. Table 1 shows the affinity measured by SPR, as well as avidity estimated by BLI. Representative SPR and BLI sensorgrams, including the association and dissociation phases, are provided in the Supplementary Materials, Figure S2. The K_D_ measured using an anti-IgG chip resulted in similar single-digit nM K_D_ values for UDIZ-007 DS-nGMP and pembrolizumab used as reference. By BLI, the K_D_ values for both UDIZ-007 DS-nGMP and pembrolizumab were three orders of magnitude lower than the SPR measurements, indicating a strong cooperative binding effect (avidity).

In addition, the K_D_ was estimated using a Protein A capture format yielding a K_D_ of 16.35 nM for UDIZ-007 DS-nGMP, compared with 2.47 nM obtained using anti-human IgG capture (Supplementary Materials, Figure S2). This difference is expected since the heavy-chain variable region of UDIZ-007 is encoded by the IGHV3-23 gene, which belongs to the VH3 family known to bind Protein A [15]. The observed difference reflects capture-surface-dependent effects, potentially affecting antibody orientation and paratope accessibility on the sensor surface, as reported by Kamat et al. [16]. 

**Figure 1:**
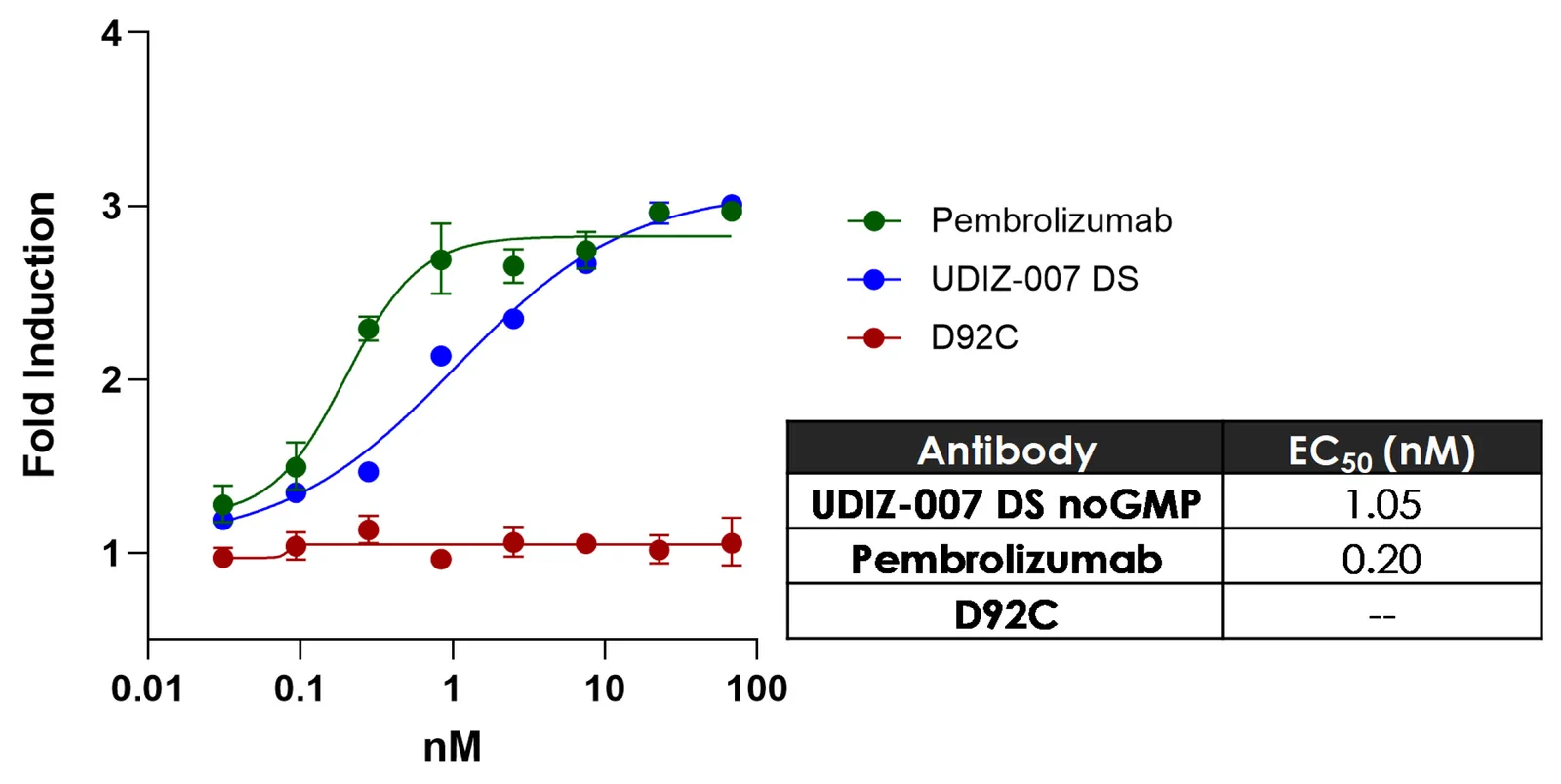
Blocking of the hPD-1/PD-L1 interaction in a cell-based assay.

The ability of UDIZ-007 DS-nGMP to block the hPD-1/PD-L1 interaction is shown in Figure 1. UDIZ-007 DS-nGMP effectively blocked hPD-1/PD-L1 binding, with an EC_50_ of 1.05 nM, whereas the EC_50_ of pembrolizumab was 0.20 nM. The five-fold difference between UDIZ-007 DS-nGMP and pembrolizumab could be due to different binding mechanisms of UDIZ-007 DS-nGMP to hPD-1 with respect to pembrolizumab (discussed below).

### 2.2. Anti-Tumor Efficacy of UDIZ-007 in a B-hPD-1 Mouse Model

**Figure 2:**
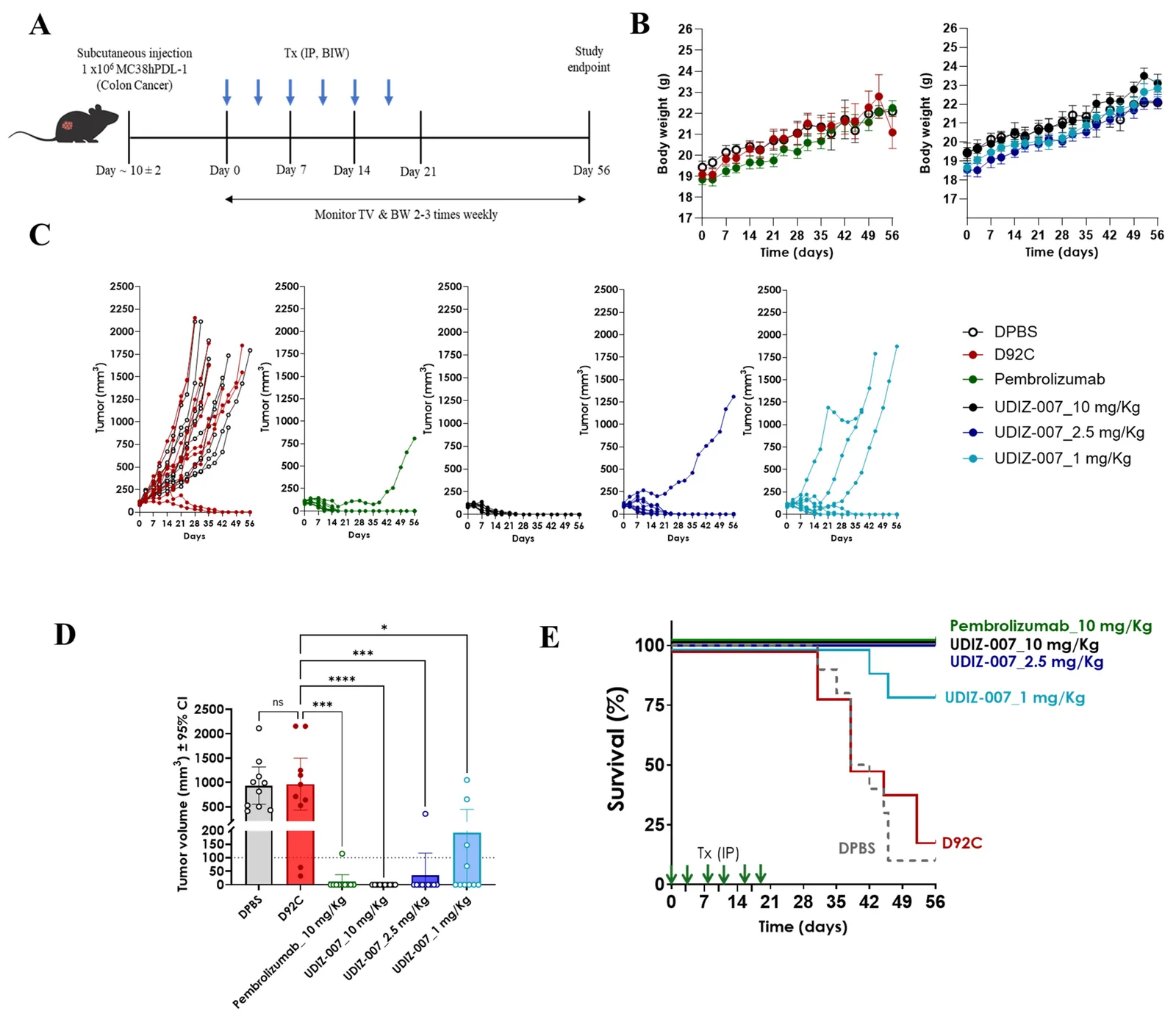
Anti-tumor efficacy of UDIZ-007 DS-nGMP in the MC38-hPD-L1 tumor model in B-hPD-1 transgenic mice. B-hPD-1 transgenic mice bearing established MC38-hPD-L1 tumors were treated with vehicle, isotype control antibody, pembrolizumab, or UDIZ-007 DS-nGMP at 1, 2.5, or 10 mg/kg according to the indicated dosing schedule. Treatment groups are consistently color-coded across panels B–E as indicated in the figure key: DPBS (gray), D92C (red), pembrolizumab (green), UDIZ-007 10 mg/kg (black), UDIZ-007 2.5 mg/kg (dark blue), and UDIZ-007 1 mg/kg (light blue) (**A**) Schematic representation of the experimental design. Treatments were administered on days 0, 3, 7, 10, 14, and 17 after treatment initiation (blue arrows). (**B**) Mean body weight profiles during the study period. (**C**) Tumor growth curves during treatment and follow-up. (**D**) Statistical comparison of tumor volume measurements across treatment groups. The data are presented as the mean ± SEM and each dot represent a TV of an individual animal on Day 28. Dotted line represents the baseline TV. (**E**) Kaplan–Meier survival analysis throughout the extended follow-up period. Solid lines represent treatment groups, the gray dashed line represents DPBS, and green arrows indicate treatment administration (Tx, IP). Survival curves were compared using the log-rank (Mantel–Cox) test. The isotype control consisted of a chimeric antibody containing anti-lysozyme D1.3 variable regions and a human IgG1 LALA isotype. Statistical significance in panel D was determined using the Kruskal–Wallis test followed by Dunn’s multiple comparisons test. * *p* < 0.05, *** *p* < 0.001, **** *p* < 0.0001, and ns (not significant) versus the isotype control group.

The anti-tumor efficacy of UDIZ-007 DS-nGMP was evaluated using MC38-hPD-L1 colon cancer cells implanted in B-hPD-1 transgenic mice. Mice were treated with vehicle, isotype control antibody, pembrolizumab, or UDIZ-007 DS-nGMP at 1, 2.5, or 10 mg/kg according to the study design shown in Figure 2A. UDIZ-007 DS-nGMP was generally well tolerated throughout the study, with no significant body weight loss (Figure 2B) or adverse clinical observations detected in any treatment group.

Tumor growth curves are shown in Figure 2C. On day 28, corresponding to the last time point at which all animals remained on study, mean tumor volumes (TVs) were 936.29 ± 168.74 mm^3^ in vehicle-treated animals, 964.61 ± 235.33 mm^3^ in the isotype control group, and 11.51 ± 11.51 mm^3^ in pembrolizumab-treated animals (Figure 2C). Mean tumor volumes in the 1, 2.5 and 10 mg/kg UDIZ-007 DS-nGMP groups were 192.27 ± 115.07 mm^3^, 35.77 ± 35.77 mm^3^ and 0.00 ± 0.00 mm^3^, respectively (Figure 2C). Corresponding %TGI (tumor growth inhibition) values relative to the isotype control group were 89.33%, 100% and 100% for the 1, 2.5 and 10 mg/kg UDIZ-007 DS-nGMP groups, respectively (Table 2). Additionally, pembrolizumab (10 mg/kg) and the 2.5 and 10 mg/kg UDIZ-007 DS-nGMP groups demonstrated tumor regression (%TR) values of 88.49%, 64.23% and 100%, respectively. The anti-tumor activity observed in the 2.5 and 10 mg/kg UDIZ-007 DS-nGMP groups was comparable to that achieved with pembrolizumab (10 mg/kg), which also demonstrated 100% TGI, confirming the responsiveness of the model to PD-1 pathway blockade. Statistical comparison of tumor volumes on Day 28 demonstrated significant anti-tumor activity for all treatment groups compared with the isotype-treated control group (Figure 2D).

Complete responses began to emerge at approximately day 17 in the 10 mg/kg UDIZ-007 DS-nGMP group and persisted through the end of the extended follow-up period (day 56). During follow-up, animals receiving 2.5 mg/kg UDIZ-007 DS-nGMP maintained sustained tumor control, with efficacy remaining comparable to the pembrolizumab-treated group. Anti-tumor responses were observed in 100%, 90%, and 70% of animals treated with the 10, 2.5 and 1 mg/kg UDIZ-007 DS-nGMP groups, respectively, compared with 90% in the pembrolizumab-treated group (Table 2).

No tumor relapse was observed in animals achieving complete responses during the extended observation period. Consistent with these findings, UDIZ-007 treatment prolonged survival relative to vehicle- and isotype-treated animals, with survival outcomes in the mid- and high-dose groups remaining comparable to pembrolizumab-treated animals throughout the extended follow-up period (Figure 2E).

Together, these findings demonstrate that UDIZ-007 DS-nGMP induces potent and durable dose-dependent anti-tumor activity in B-hPD-1 mice bearing MC38-hPD-L1 tumors and supports effective blockade of the human PD-1/PD-L1 pathway in vivo. The persistence of complete responses after treatment discontinuation further suggests sustained immune-mediated tumor control following PD-1 pathway inhibition.

### 2.3. Single-Dose Pharmacokinetic Study in Mice

**Figure 3:**
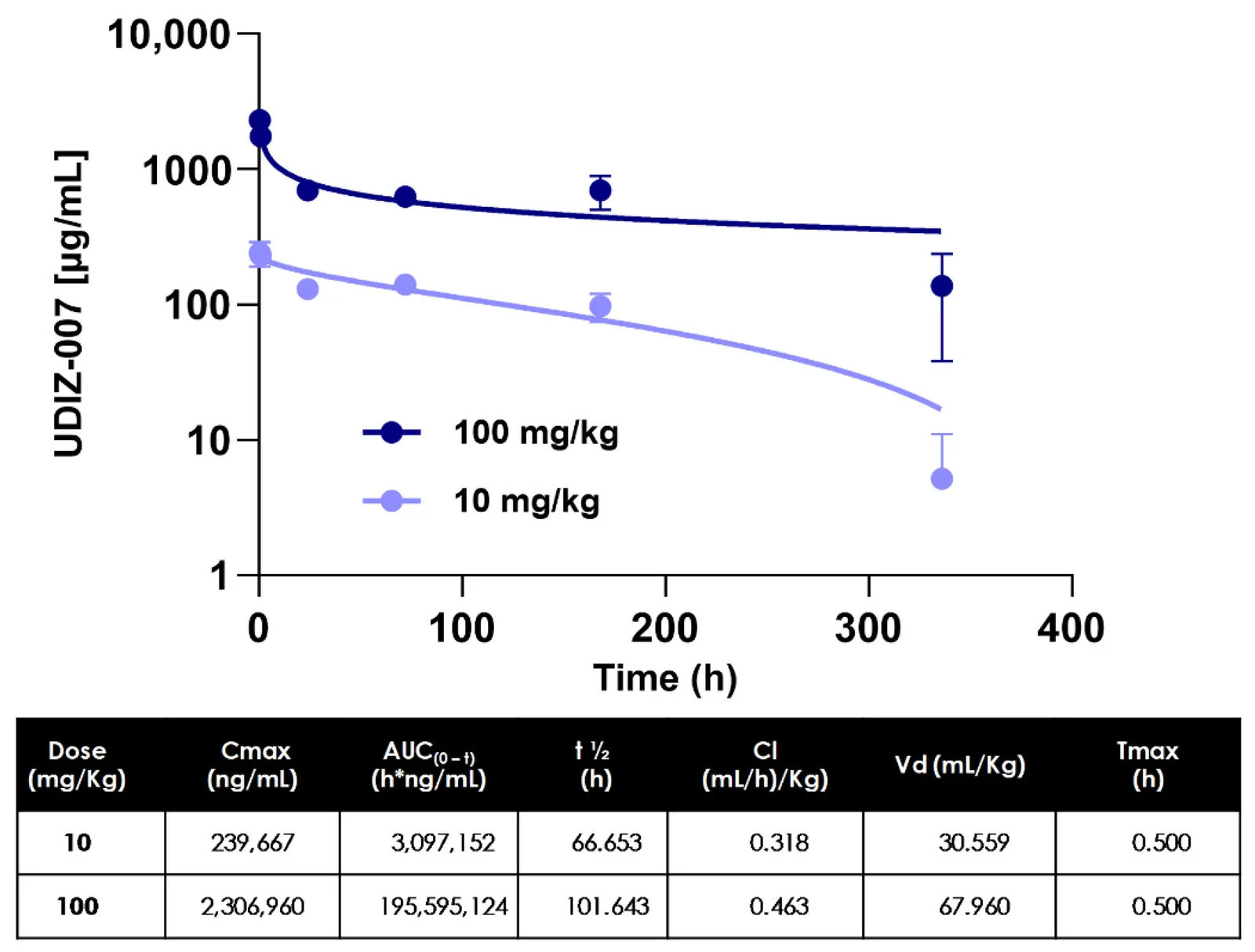
PK profile of UDIZ-007 DS-nGMP at a single IV bolus administration of 10 and 100 mg/kg in B-hPD-1 transgenic mice.

The PK of UDIZ-007 DS-nGMP following a single IV bolus administration of 10 and 100 mg/kg in B-hPD-1 transgenic mice is shown in Figure 3. Serum concentration–time profiles demonstrated a biphasic decline characteristic of human or humanized monoclonal antibodies administered in mice [17].

The maximum serum concentration (Cmax) was observed at the first sampling time point (0.5 h post-dose) for both dose levels, with mean values of 239.7 μg/mL and 2307.0 μg/mL for the 10 and 100 mg/kg groups, respectively. Systemic exposure, measured as the area under the curve (AUC), increased in a dose-proportional manner, as the dose-normalized AUC values were comparable between the two groups (3146.7 vs. 2157.7 h·kg·μg/mL/mg). The terminal half-life (t_1_/_2_) was 66.7 h (2.8 days) at 10 mg/kg and almost twice as long (101.6 h; 4.2 days) at 100 mg/kg. Systemic clearance (Cl) remained low at both dose levels (0.318 and 0.463 mL/h/kg, respectively), while the apparent volume of distribution (Vd) ranged from 30.6 to 68.0 mL/kg. The longer half-life observed at the higher dose level may suggest partial saturation of target-mediated clearance pathways. Treatment was well tolerated, with no significant body weight loss or adverse clinical signs observed during the 14-day study period.

### 2.4. Single-Dose Pharmacokinetic Study in Cynomolgus Macaques

**Figure 4:**
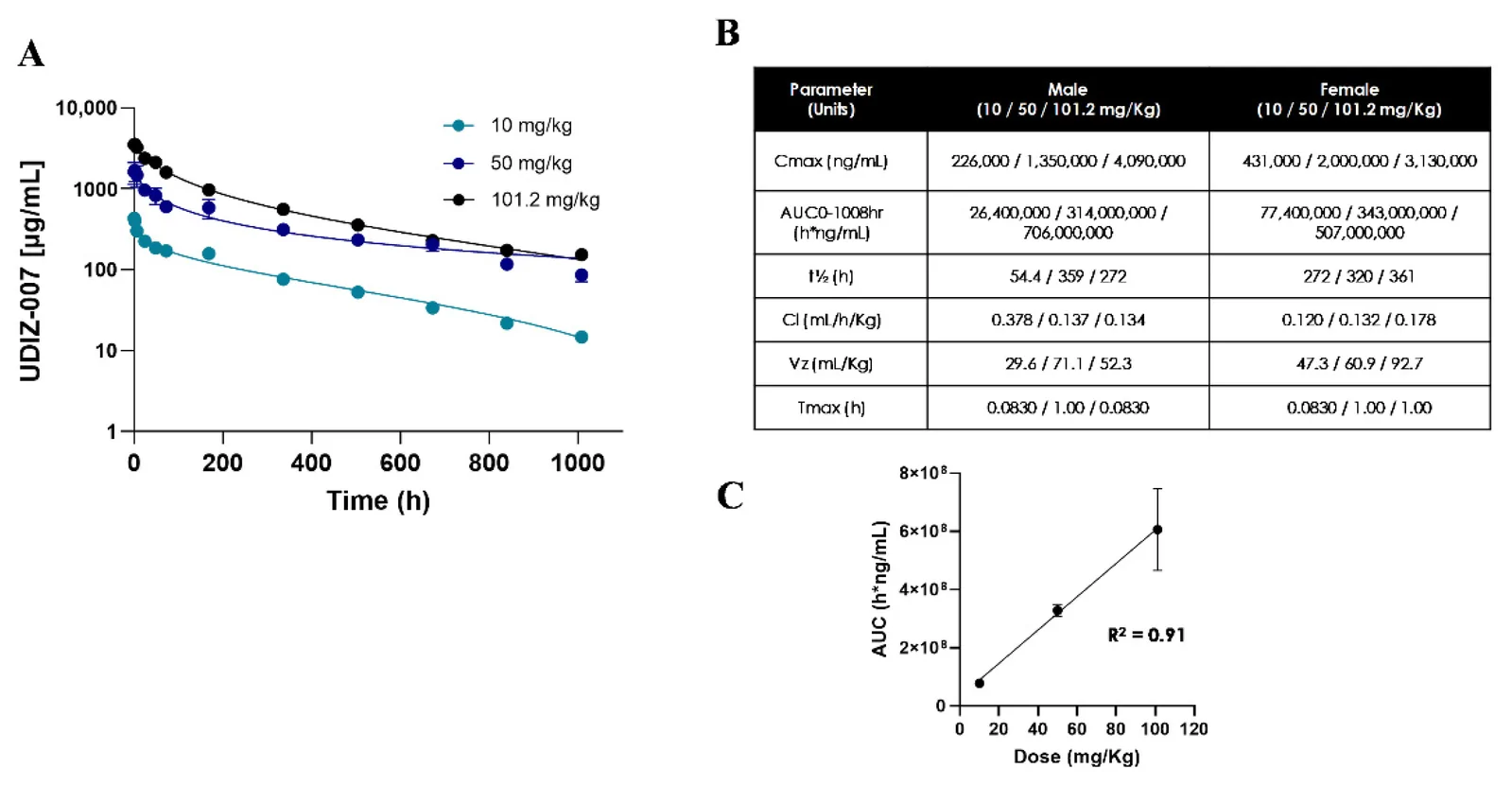
Cynomolgus macaque PK of UDIZ-007 DS-nGMP at single IV doses of 10, 50, and 101.2 mg/kg. (**A**) Kinetics of UDIZ-007 DS-nGMP serum concentration at the three dose levels. (**B**) Main PK parameters. (**C**) Comparison of AUC_0-1008_ for three dose levels of UDIZ-007 DS-nGMP.

In cynomolgus macaques, UDIZ-007 DS-nGMP PK analysis following single IV doses of 10, 50, and 101.2 mg/kg showed a Cmax at the first sampling time points (0.083–1 h post-dose) that was proportional to the dose (Figure 4A). However, this proportionality was not observed in the male subject dosed at 10 mg/kg group, whose pharmacokinetic values were atypical relative to both the female in the same cohort and the overall dose–response trend seen in the other groups (Figure 4B).

The male receiving the dose of 10 mg/kg was excluded from the analysis, as it developed ADAs from time point 336 h (Supplementary Material, Table S1). For the rest of the animals, which remained ADA-negative throughout the entire sampling period, the t_1_/_2_ ranged from 272 to 361 h (11.3–15.1 d), with low systemic clearance (Cl: 0.120–0.178 mL/h/kg), consistent with a slow elimination of human monoclonal antibodies in cynomolgus macaques (Figure 4B). The AUCs computed for UDIZ-007 increased proportionally with the dose (Figure 4C). Collectively, these findings demonstrate sustained systemic exposure and predictable pharmacokinetic behavior of UDIZ-007 DS-nGMP following IV administration.

### 2.5. Toxicokinetic and Toxicologic Study in Cynomolgus Macaques

**Figure 5:**
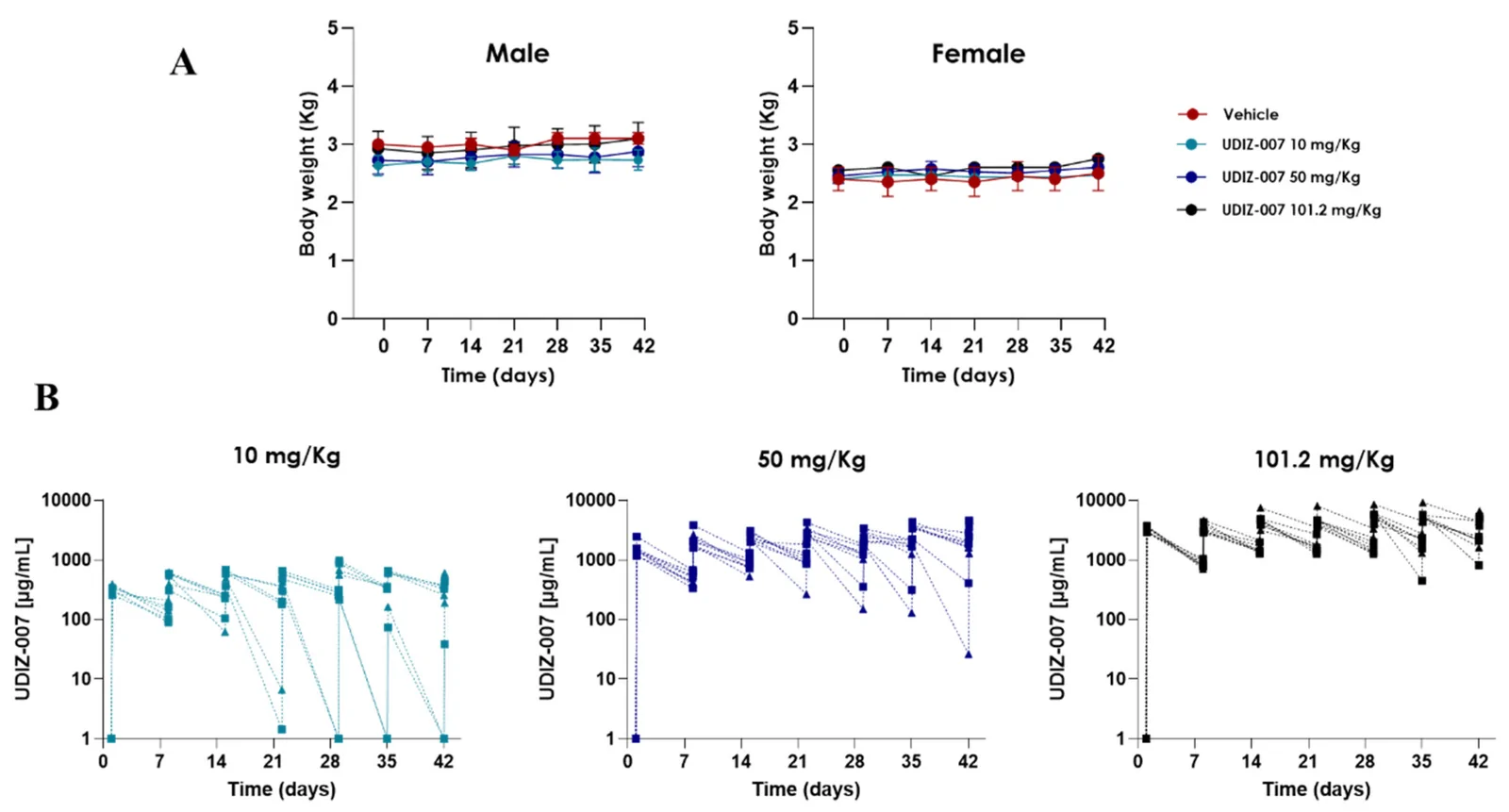
Cynomolgus macaque TK of UDIZ-007 DS-nGMP at repeated IV administrations of 10, 50, or 101.2 mg/kg once a week for six consecutive weeks. (**A**) Body weight. (**B**) Individual TK at the three doses (triangles, females; squares, males).

We also evaluated the toxicokinetic and systemic toxicity profile of UDIZ-007 DS-nGMP in the repeated-dose study receiving UDIZ-007 DS-nGMP IV once weekly (10, 50, or 101.2 mg/kg) for six consecutive weeks, followed by a four-week recovery phase. Body weights were comparable across groups throughout the dosing and recovery periods (Figure 5A).

Serum concentrations of UDIZ-007 DS-nGMP remained detectable throughout the repeated-dose phase for most animals. On day 42, 13 animals receiving UDIZ-007 DS-nGMP were ADA-positive: 5/6 for 10 mg/kg, 4/8 for 50 mg/kg, and 4/8 for 101.2 mg/kg (Supplementary Material, Table S2). In the vehicle-treated group, 2/4 animals were ADA-positive, suggesting that positivity was unrelated to UDIZ-007 DS-nGMP administration. Furthermore, 10/13 of the ADA-positive macaques had serum concentration < 4 S:N, indicating a low ADAs response in most macaques. The three remaining ADA-positive animals had serum levels above 94 S:N. Those high ADA levels correlated with reductions in drug exposure during the repeat-dose phase and were, therefore, excluded from the TK analysis.

Weekly administration of UDIZ-007 DS-nGMP produced Cmax and Ctrough that were dose-dependent throughout the analyzed period. However, the dose schedules were not enough to reach the steady state in serum concentrations. This was expected as the maximum follow-up time was approximately three t1/2. Thus, we did not calculate Cmax/ Cmin at steady state. However, total exposure of UDIZ-007 DS-nGMP increased proportionally with the dose (Figure 5B), in agreement with the PK analysis performed after single-dose administration.

To correlate the drug exposure with the appearance of toxic effects, toxicology analysis was performed in the same animals. The toxicology findings are summarized in Table 3. Briefly, no mortality or treatment-associated clinical signs were observed during the study; body weights were comparable across groups throughout the dosing and recovery periods; ophthalmologic examinations, electrocardiographic assessments, hematology, coagulation, clinical chemistry, urinalysis, organ weights, and gross pathology evaluations did not reveal toxicity attributable to UDIZ-007 DS-nGMP administration. Furthermore, while a fraction of animals developed ADAs, no impact of the ADAs was observed on clinical or pathological analyses.

Histopathological evaluation identified mononuclear cell infiltrates within the choroid plexus in a limited number of animals at the 50 and 101.2 mg/kg/dose levels. The incidence and severity distribution of choroid plexus mononuclear cell infiltrates occurred in the 50 mg/kg/dose group in 1 out of 3 males (mild severity) and in 0 out of 3 females. In the 101.2 mg/kg/dose group, infiltrates were observed in one out of three males (moderate severity) and in one out of three females (mild severity). No infiltrates were observed in vehicle control animals of either sex. No infiltrates were observed in any recovery euthanasia animals, indicating reversibility. The degree of those infiltrates ranged from mild to moderate and was not associated with neurological manifestations. Importantly, these histopathological observations were absent in macaques following the 28-day recovery period, indicating reversibility. Because PD-1 blockade enhances immune activation, and the infiltration was reversible after the recovery period, these findings are more likely to reflect localized immune-mediated effects rather than nonspecific tissue toxicity.

Based on the overall safety profile, including the absence of mortality, lack of UDIZ-007 DS-nGMP-related effects on clinical observations, body weight, ophthalmology, ECG, clinical pathology, organ weights, and macroscopic findings, and the reversibility of the only microscopic finding (choroid plexus infiltrates), the NOAEL was determined to be 101.2 mg/kg, the highest dose tested. At the NOAEL, the Cmax and AUC_0-1008hr_ were determined to be 3,130,000 and 4,090,000 ng/mL and 507,000,000 and 706,000,000 h*ng/mL for females and males, respectively, following a single administration on day 1.

### 2.6. Human and Cynomolgus Macaque Tissue Cross-Reactivity

**Figure 6:**
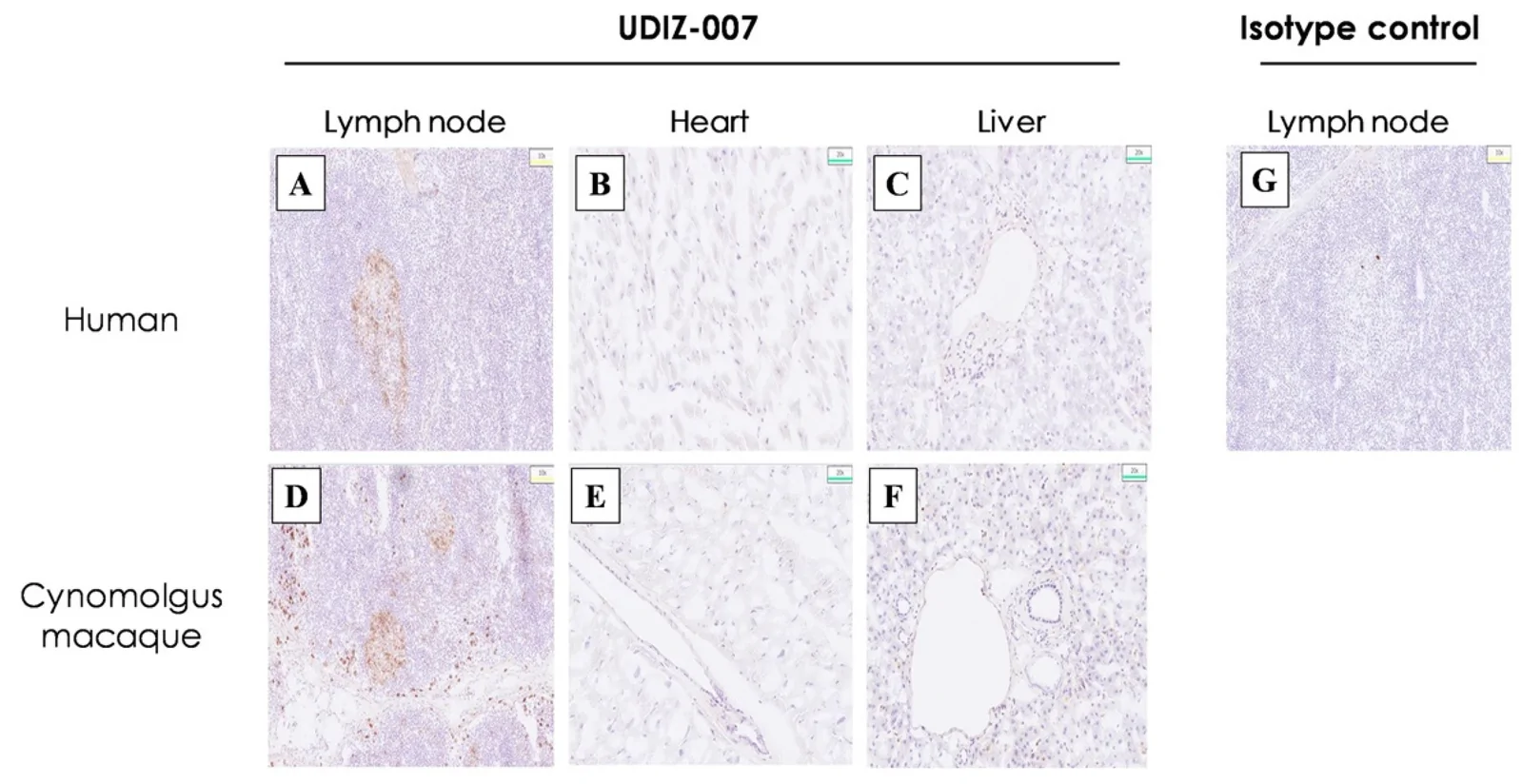
Representative tissue cross-reactivity profile of UDIZ-007 in human and cynomolgus macaque tissues. (**A**) Human lymph node demonstrating membranous and cytoplasmic staining of lymphocyte populations within lymphoid regions. (**B**) Human heart showing absence of specific staining in non-lymphoid parenchymal tissue. (**C**) Human liver showing no specific binding in hepatic parenchyma. (**D**) Cynomolgus macaque lymph node showing a lymphocyte-restricted staining pattern comparable to that observed in human lymphoid tissue. (**E**) Cynomolgus macaque heart showing absence of specific staining in cardiac tissue. (**F**) Cynomolgus macaque liver demonstrating no specific parenchymal staining. (**G**) Human lymph node incubated with isotype control antibody demonstrating absence of nonspecific background staining. Staining was restricted primarily to lymphocyte populations within lymphoid tissues, while no specific staining was observed in non-lymphoid parenchymal tissues. Scale bars shown in inserts.

The tissue cross-reactivity (TCR) profile of UDIZ-007 DS-nGMP was evaluated via immunohistochemistry using a broad panel of normal human and cynomolgus macaque tissues to assess binding specificity and potential off-target reactivity. Representative staining patterns are shown in Figure 6, and a comprehensive summary of the tissue cross-reactivity dataset is provided in the Supplementary Material, Table S3. UDIZ-007 DS-nGMP showed concentration-dependent binding that was restricted primarily to lymphocyte populations within lymphoid tissues, including lymph node, spleen, thymus, tonsil, and gut-associated lymphoid tissue. The observed staining pattern was predominantly membranous and cytoplasmic, consistent with the known distribution of PD-1-expressing activated immune cells.

Importantly, no specific binding to parenchymal or non-lymphoid tissues was identified across cardiovascular, pulmonary, hepatic, renal, neurologic, gastrointestinal, reproductive, placental, muscular, or cutaneous tissues. Similar staining patterns were observed between human and cynomolgus macaque tissues. Overall, these findings demonstrate that UDIZ-007 DS-nGMP exhibits a highly selective tissue binding profile consistent with the expected physiological distribution of PD-1, without evidence of unexpected off-target tissue reactivity.

## 3. Discussion

Here, we reported the preclinical characterization of the fully human anti-PD-1 antibody UDIZ-007 produced in a high-yield stable CHO cell line. The biophysical profile of UDIZ-007 DS-nGMP was consistent with that expected for therapeutic antibodies, as well as with our previous report [14], which used research-grade UDIZ-007 transiently expressed in CHO cells. The affinity of UDIZ-007 DS-nGMP for hPD-1 measured by SPR using an anti-human IgG capture format was in the single-digit nanomolar range. This K_D_ value falls within the range of affinities reported for other anti-PD-1 antibodies clinically validated for human therapy, such as pembrolizumab, nivolumab, cemiplimab and camrelizumab [7]. The avidity of UDIZ-007 DS-nGMP assessed by BLI was in the low picomolar range, approximately three orders of magnitude stronger than the monovalent affinity measurement. It was also comparable to that of pembrolizumab analyzed side by side with UDIZ-007 DS-nGMP.

Despite a similar affinity and avidity, the hPD-1/PD-L1 blocking activity of UDIZ-007 DS-nGMP in a cell-based assay was five-fold lower than that observed for pembrolizumab. However, it has been reported that the affinity for hPD-1 does not necessarily correlate with the hPD-1/PD-L1 blocking potency of anti-PD-1-therapeutic antibodies [7], as the blocking activity also depends on the specific epitope recognized by the antibody and the mechanism by which the antibody engages hPD-1. The UDIZ-007 epitope, as determined by cross-linking mass spectrometry (XL-MS), is located in a region comprising the C’D loop, *β*-strands C and C’, FG loop and *β*-strand E [14]; the latter has not been reported as part of the epitopes recognized by any other anti-PD-1 antibody of known structures in complex with hPD-1 [7]. In fact, pembrolizumab binds hPD-1 at the BC, *β*-strands C and C’, and CC’ and FG loops [18], suggesting a different orientation and mode of engagement with PD-1 as compared to UDIZ-007. Although the fine details of UDIZ-007 interaction with hPD-1, relative orientation of the antibody with respect to hPD-1 and potential conformational changes upon binding, remain to be confirmed by the structure of the hPD-1:UDIZ-007 complex determined with a higher-resolution method such as X-ray crystallography, the difference in the epitope location of UDIZ-007 with respect to pembrolizumab could account for the difference in the blocking potency observed in the two antibodies.

Further, although UDIZ-007 DS-nGMP exhibited lower hPD-1/PD-L1 in vitro blocking activity than pembrolizumab, this did not appear to impact its anti-tumor efficacy in vivo. UDIZ-007 DS-nGMP demonstrated strong anti-tumor activity in the MC38-hPD-L1 B-hPD-1 model, with efficacy comparable to that observed with pembrolizumab. Notably, comparable anti-tumor activity was achieved with UDIZ-007 DS-nGMP at 2.5 mg/kg and pembrolizumab at 10 mg/kg, indicating robust efficacy of UDIZ-007 DS-nGMP despite the lower administered dose. Tumor growth inhibition was observed early after treatment initiation and was accompanied by dose-dependent increases in tumor regression, complete response rate, and survival. These findings are consistent with the restoration of endogenous anti-tumor immune responses following PD-1 pathway inhibition. Importantly, complete responses persisted after treatment discontinuation, suggesting durable immune-mediated tumor control rather than a transient pharmacologic effect. In addition, treatment did not affect body weight or produced overt signs of toxicity, indicating that substantial anti-tumor activity was achieved without evidence of major systemic intolerance under the conditions evaluated. Moreover, anti-tumor efficacy was evaluated using female B-hPD-1 transgenic mice in accordance with the validated protocol for this established model. Consequently, the study was designed to compare the efficacy of UDIZ-007 DS-nGMP and pembrolizumab under standardized experimental conditions and was not intended or statistically powered to evaluate sex-specific differences in treatment response. 

Importantly, the overall non-clinical package included PK and repeated-dose toxicology studies in both male and female cynomolgus macaques, providing safety and pharmacokinetic data from both sexes. Single-dose PK studies in cynomolgus macaques demonstrated dose-proportional exposure, prolonged half-life, and low clearance, consistent with the expected PK behavior of IgG-based therapeutic antibodies [19]. The t_1/2_ of UDIZ-007 DS-nGMP ranged from 272 to 361 h across most dose groups, which falls within the range reported for other fully human anti-PD-1 therapeutic antibodies [20,21]. For example, the values reported for nivolumab are 124–139 h at 1 mg/kg and 261 h for 10 mg/kg [20]. These similarities are expected given that both antibodies share the same binding targets and display comparable affinities. Although these data should be viewed as exploratory and hypothesis-generating, systemic exposure increased proportionally with increasing dose levels across the evaluated range, indicating that the antibody does not show non-linear PK, thus supporting predictable dose-dependent PK. 

These results were confirmed in a second study with repeated IV administration of UDIZ-007 DS-nGMP. Repeated IV administrations of UDIZ-007 DS-nGMP were well tolerated, with no major treatment-related toxicities observed, even at the higher dose of 101.2 mg/kg. Thus, the NOAEL was set at 101.2 mg/kg. The strategy to calculate the NOAEL identified the highest dose level at which no significant increase in adverse effects is observed compared with the control group after seven weekly administrations of UDIZ-007 in male and female cynomolgus macaques. Doses higher than 101.2 mg/kg were not tested in accordance with ICH S6(R1) and ICH S9 regulatory guidelines [22,23], since the highest dose tested already achieved systemic exposure substantially exceeding the anticipated human therapeutic range of anti-PD-1 antibodies.

The therapeutic dose of Keytruda^®^ is ~3 mg/kg, and 101.2 mg/kg substantially exceeds (~40x) it. We tested 1, 2.5 and 10 mg/kg for UDIZ-007 in the efficacy model in this work and 10 mg/kg in a previous report [14]. At 10 mg/kg, we observed total elimination of the tumors, and 101.2 mg/kg is ~10x this dose. At 2.5 mg/kg, significant anti-tumor activity (similar to Keytruda^®^) was observed, again substantially exceeding (~40x) the anticipated human therapeutic dose. This risk prediction strategy is appropriate for immunomodulatory biotechnological drugs intended for oncology applications when the drug (1) has a well-defined mechanism of action (MOA); (2) shares the same MOA as other products with established clinical experience demonstrating that they are not high-risk; and (3) has undergone in vivo safety evaluations in relevant animal models whose results do not suggest toxicity [24]. The conversion to the human equivalent dose (HED) considers a scaling factor of 1.0, as recommended by the FDA for biotechnological drugs with a molecular weight greater than 100 kDa administered via the intravascular route [25]; therefore, the HED is also established at 101.2 mg/kg.

The only treatment-related finding consisted of reversible mononuclear cell infiltrates within the choroid plexus at the mid- and high-dose levels. Because these infiltrates resolved during the recovery phase and PD-1 blockade is expected to enhance immune activation, these findings can be interpreted as localized transient immune-mediated effects rather than with nonspecific tissue injury. Together, these results support the potential of UDIZ-007 DS-nGMP as an immunotherapeutic agent capable of inducing durable anti-tumor immune responses through PD-1 pathway blockade with no major treatment-related toxicities, even at the higher dose of 101.2 mg/kg.

The detection of ADAs in multiple animals was also consistent with the immunogenicity reported for other fully human anti-PD-1 therapeutic antibodies in non-human primates [20,21,26] and did not correlate with adverse clinical or pathological findings associated with UDIZ-007 DS-nGMP administration. As a reference, preclinical studies with nivolumab [20] reported ADAs in approximately half of the low-dose animals (1 mg/kg), as well as in isolated animals in mid- and high-dose groups. A similar inverse relationship between dose level and ADA incidence was observed in our studies. One possible explanation for this phenomenon, also discussed for nivolumab, is that high circulating antibody concentrations interfere with ADA detection in bridging assays by saturating available binding sites required for assay signal generation. Because the ADA assay used in this study relied on competitive binding between circulating UDIZ-007 and the labelled detection antibody, high drug concentrations may have reduced the sensitivity for ADA detection in animals receiving higher doses. Similar assay-related limitations have been described previously for therapeutic antibodies in non-human primate studies [27,28].

It is also important to consider that UDIZ-007 is a fully human antibody administered to cynomolgus macaques, and some degree of immunogenicity is, therefore, expected in this species [26]. Although the cynomolgus macaque remains the most relevant non-clinical species for evaluating PK and safety of PD-1-targeting antibodies, ADA responses observed in non-human primates do not necessarily predict the incidence or clinical impact of immunogenicity in humans [26,29].

Finally, human and cynomolgus macaque tissue cross-reactivity showed that UDIZ-007 binding was largely restricted to lymphocyte populations within lymphoid tissues, consistent with the expected distribution of PD-1-expressing activated immune cells. No specific staining was observed in non-lymphoid or parenchymal tissues, including cardiovascular, neurologic, hepatic, renal, pulmonary, reproductive, or placental tissues, suggesting minimal off-target tissue binding. Similar staining patterns were observed across human and cynomolgus macaque tissues, corroborating the relevance of cynomolgus macaques for non-clinical safety studies. Overall, these findings indicate that UDIZ-007 DS-nGMP displays a selective tissue staining profile consistent with the expected distribution of PD-1-targeted antibodies.

In summary, UDIZ-007 DS-nGMP: (1) induced dose-dependent anti-tumor activity; (2) showed a trend toward dose-proportional exposure together with a prolonged half-life consistent with therapeutic monoclonal antibodies; (3) Repeated IV administrations of 10, 50 and 101.2 mg/kg were well tolerated, with no major treatment-related toxicities identified; and (4) tissue cross-reactivity studies in human and cynomolgus macaques demonstrated a staining pattern restricted to lymphoid tissues known to express PD-1. Together, these results support the efficacy and safety of UDIZ-007 to advance this antibody-based drug to clinical development.

## 4. Materials and Methods

### 4.1. Cell Line Development, Purification, and Characterization of UDIZ-007 DS-nGMP

The antibody preparation used in this study, designated UDIZ-007 DS-nGMP, was produced at ProBio (Hopewell, NJ, USA) as follows. A high-yield stable Chinese Hamster Ovary (CHO) cell line was generated using standard cloning and selection procedures, followed by establishment of a master cell bank. Expression was scaled up to a 50 L bioreactor, and the antibody was purified from cell culture supernatants by Protein A affinity chromatography using a MabSelect PrismA column (Cytiva, Cat. No. 17549803, Uppsala, Sweden), followed by anion-exchange chromatography using a POROS XQ column (Thermo Fisher Scientific, Cat. No. 4467818, Chelmsford, MA, USA) and multimodal cation-exchange chromatography using CHT Type II resin (Bio-Rad, Cat. No. 157-4500, Hercules, CA, USA). Final concentration and buffer exchange were performed by ultrafiltration/diafiltration. The final product (UDIZ-007 DS-nGMP) was formulated in 10 mM His-HCl buffer, pH 6.0, containing sucrose and polysorbate 80, followed by sterile filtration and aliquoting.

The physicochemical properties of UDIZ-007 DS-nGMP were evaluated by SDS-PAGE, analytical size-exclusion chromatography (SEC-UPLC), and intact mass analysis by mass spectrometry (MS), as previously described by Gomez-Castellano et al. [14]. In addition, peptide mapping by mass spectrometry was performed under both reducing and non-reducing conditions to confirm the primary structure and disulfide bond connectivity of UDIZ-007 DS-nGMP. For reducing peptide mapping, antibody samples were reduced with dithiothreitol (DTT, 5 mM), alkylated with iodoacetamide (0.6 M), and denatured with 3.75 M guanidine hydrochloride at 80 °C. Samples were subsequently digested with sequencing-grade trypsin at 37 °C for 18 h. For non-reducing peptide mapping, samples were denatured under identical conditions without reduction or alkylation prior to trypsin digestion. After digestion, reactions were quenched with formic acid, and peptide mixtures were filtered through 0.2 μm membranes before analysis. Peptide analyses were performed using a Vion^®^ hybrid mass spectrometer (Waters, ESI-IMS-Q-ToF, Wilmslow, UK) coupled to an ACQUITY UPLC Class I system (Waters, Milford, MA, USA) equipped with a Peptide CSH C18 column (2.1 × 150 mm, 1.7 μm). Peptides were separated using a gradient of acetonitrile/water containing 0.1% formic acid, and data were processed using UNIFI^®^ software version 1.9.4.053 (Waters).

### 4.2. Binding to hPD-1 by SPR and PD-1/PD-L1 Blocking Activity in Vitro

The in vitro functionality of UDIZ-007 DS-nGMP was evaluated at three levels: (1) binding affinity to human PD-1 (hPD-1) by surface plasmon resonance (SPR), (2) avidity toward hPD-1 by biolayer interferometry (BLI), and (3) hPD-1/PD-L1 blocking activity in a cell-based assay. As a reference control, Keytruda^®^ (pembrolizumab) was evaluated side by side with UDIZ-007 DS-nGMP.

SPR was performed by using a Biacore T200 instrument (Cytiva^®^, Uppsala, Sweden) with a goat anti-human Fc polyclonal antibody (R&D Systems, Cat. No. G-102-C; Minneapolis, MN, USA) immobilized onto a CM5 sensor chip (Cytiva^®^ Cat. No. BR100399, Uppsala, Sweden) using an amine coupling kit according to the manufacturer’s instructions. UDIZ-007 DS-nGMP was diluted in HBS-EP 1x buffer, pH 7.4 (Cytiva^®^, Marlborough, MA, USA), and captured onto the sensor surface at 25 °C using a flow rate of 10 µL/min and a contact time of 30 s. Recombinant hPD-1 (Acro Biosystems Cat. No. PD-1-H522a, Newark, DE, USA) diluted in HBS-EP buffer was subsequently injected in a range of concentrations of 6.25–50 nM using a flow rate of 10 µL/min, an association time of 150 s, and a dissociation time of 300 s. Sensorgrams were analyzed using a 1:1 Langmuir binding model with BIAevaluation software version 3.1 (Cytiva^®^).

Assessment of the cooperative binding effect (avidity) of UDIZ-007 DS-nGMP by BLI was conducted in the Octet RED96 instrument (Sartorius Corporation, Bohemia, NY, USA). HIS1K biosensors (Sartorius, Cat. No. 18-5120, Fremont, CA, USA) were loaded with recombinant PD-1-His protein (GenScript, Cat. No. Z03424-100, Nanjing, Jiangsu, China) at 5 μg/mL for 300 s. Baseline, loading, association, and dissociation steps were performed in 1% BSA in 0.02% PBS Tween-20 at a shake speed of 1000 rpm. Serial dilutions of UDIZ-007 DS-nGMP ranging from 1.56 to 100 nM were evaluated in kinetic binding assays with association and dissociation times of 90 s. Sensor regeneration was performed using 10 mM glycine-HCl, pH 1.5. Data acquisition was performed using standard kinetics settings (5.0 Hz), and binding kinetics were analyzed using Octet^®^ System Data Analysis software version 10.0.

PD-1/PD-L1 blocking activity of UDIZ-007 DS-nGMP was evaluated as previously described [14]. In brief, recombinant CHO-K1 cells constitutively expressing hPD-L1 (PD-L1/TCR Activator CHO Recombinant cell line, BPS Bioscience, Cat. No. 60536, San Diego, CA, USA) (3.5 × 10^4^ cell/well) were cultivated on Thaw Medium 3A (BPS Bioscience Cat. No. 60186, San Diego, CA, USA) overnight at 37 °C/5% CO_2_. In parallel, increasing concentrations of UDIZ-007 and control antibodies from 5 × 10^−4^ to 10 µg/mL were pre-incubated for 30 min at 37 °C/5% CO_2_ with PD-1/NFAT reporter Jurkat recombinant cells (4 × 10^4^ cell/well). This mix was added to the CHO-K1 cells and incubated for six hours at 37 °C/5% CO_2_. Finally, One-StepTM Luciferase assay (BPS Bioscience, Cat. No. 60690, San Diego, CA, USA) was added, and luminescence was recorded using a SpectraMax M3 microplate reader (Molecular Devices, San Jose, CA, USA).

### 4.3. Animal Husbandry and Welfare Monitoring

All animal procedures were conducted in accordance with protocols approved by the relevant Institutional Animal Care and Use Committees (IACUCs) at the performing institutions. Animals were acclimated prior to study and housed under controlled environmental conditions with species-appropriate food, water, and environmental enrichment provided according to institutional standard operating procedures and applicable welfare regulations, including the Guide for the Care and Use of Laboratory Animals.

Animals were monitored daily throughout the study period for body weight, clinical condition, and overall health status. Humane endpoint criteria included excessive body weight loss, severe clinical signs, impaired mobility, inability to access food or water, tumor ulceration, excessive tumor burden (for efficacy studies), or other conditions indicative of compromised animal welfare. Animals reaching predefined humane endpoint criteria were euthanized immediately.

### 4.4. Efficacy in a B-hPD-1 Transgenic Mouse Model

The analysis of UDIZ-007 DS-nGMP efficacy was performed by Biocytogen (Waltham, MA, USA). Female B-hPD-1 transgenic mice (C57BL/6-Pdcd1tm1(PDCD1)/Bcgen), approximately 7 weeks of age, were used in accordance with the validated Biocytogen efficacy protocol for this model. Animals were subcutaneously inoculated in the right flank with 1 × 10^6^ hPD-L1-expressing murine colon carcinoma MC38 cells (B-hPD-L1 MC38) (Biocytogen, Cat. No. 310685, Beijing, China). The B-hPD-1/MC38-hPD-L1 model was selected because it enables evaluation of antibodies targeting the human PD-1/PD-L1 pathway in an immunocompetent syngeneic setting while preserving endogenous immune cell interactions. The B-hPD-1 transgenic mice and B-hPD-L1 MC38 tumor cell line are commercially validated models developed by Biocytogen. Human PD-1 expression in the transgenic mice and stable human PD-L1 expression in the MC38 tumor cells were previously confirmed by the provider using flow cytometry during model development. Pembrolizumab was included as a positive control to confirm the responsiveness of the model to PD-1 pathway blockade. When tumors reached a mean volume of approximately 100 mm^3^ (range: 76.76–124.04 mm^3^), animals were randomly distributed into 6 groups (n = 10/group) and the treatments were initiated. Treatments included vehicle control (DPBS), D92C antibody as isotype control (10 mg/kg), pembrolizumab as positive control (10 mg/kg), and three dose levels of UDIZ-007 DS-nGMP (1, 2.5, and 10 mg/kg) diluted in DPBS. Treatments were administered intraperitoneally on days 0, 3, 7, 10, 14, and 17 after treatment initiation (six total administrations).

Tumor volumes (TVs) and body weights (BWs) were measured twice a week until day 56 post-treatment. TV was calculated using the formula TV = (W^2^ × L)/2, where “W” is the width measured along the shortest axis of the tumor, and “L” is the length measured along the longest axis. Anti-tumor efficacy was determined using two indicators: (1) tumor growth inhibition percentage (%TGI) and (2) tumor regression percentage (%TR). The %TGI was calculated using the formula %TGI = [1 − (Tt − T_0_) / (Ct − C_0_)] × 100%, where Tt is the mean TV of the treated group on day t, T_0_ is the mean tumor volume of the treated group on day zero (start of treatment), Ct is the mean tumor volume of the control group on day t, and C_0_ is the mean tumor volume of the control group on day 0. %TR was calculated using the formula %TR = [1 − (Tt/T_0_)] × 100%. A complete response (CR) was considered when no detectable tumor was present at the end of the study. Tumor volumes were assessed twice weekly; therefore, animals exceeding the predefined tumor volume endpoint at a scheduled assessment were euthanized immediately following tumor measurement in accordance with the approved IACUC protocol. Humane euthanasia criteria included tumor volume exceeding 2000 mm^3^, tumor ulceration, or body weight loss ≥20% relative to baseline measurements. Animals reaching humane endpoints or surviving to the scheduled study endpoint were euthanized by CO_2_ inhalation followed by a secondary euthanasia procedure.

### 4.5. Single-Dose Pharmacokinetic in B-hPD-1 Transgenic Mice

The PK of UDIZ-007 DS-nGMP was evaluated in female B-hPD-1 mice aged 9–10 weeks. In two groups of six mice per group, each received 10 mg/kg or 100 mg/kg. The antibody was administered by tail vein injection. Blood samples were collected at multiple time points, including pre-dose (day − 14) and at 0.5, 1, 24, 72, 168, and 336 h post-dose. At all time points, approximately 50 µL of blood was collected from the facial vein into microtubes with serum separator gel, with the exception of the terminal time point (336 h), for which approximately 500 µL of blood was collected by cardiac puncture and placed into serum separator tubes. Blood was centrifuged to obtain serum, which was immediately frozen and stored at −80 °C until analysis. Serum levels of UDIZ-007 DS-nGMP were quantified using an electrochemiluminescence immunoassay (MSD). Briefly, MSD plates were coated with an anti-human IgG capture antibody. Afterwards, blocking, standards, quality controls (QCs), and diluted serum samples were added. Detection was performed with an anti-human IgG antibody conjugated to SULFO-TAG, followed by the addition of the read buffer. Electrochemiluminescent (ECL) signals were measured using a MESO QuickPlex SQ 120 reader (Meso Scale Diagnostics, Rockville, MD, USA). PK parameters, including Cmax, Tmax, Vd, AUC_0−t_, Cl, AUC_0−∞_, and T_1_/_2_, were calculated using Certara Phoenix™ software (version 8.1). At the terminal sampling time point (336 h), animals were euthanized by CO_2_ inhalation followed by a secondary euthanasia procedure. Death was confirmed according to institutional procedures prior to terminal blood collection.

### 4.6. Single-Dose Pharmacokinetics in Cynomolgus Macaques

This study was conducted at Charles River (Fairfield, NJ, USA) using naïve cynomolgus macaques (Macaca fascicularis) approximately 29–44 months of age, divided into three groups (10, 50, and 101.2 mg/kg) of one female and one male per group. All three groups received a single IV injection as a slow bolus (3–5 min) into the cephalic vein. Blood samples were collected at the following time points: pre-dose (0 h) and at 0.083, 1, 6, 24, 48, 72, 168, 336, 504, 672, 840, and 1008 h post-dose. Blood samples were processed to obtain serum.

The concentration of UDIZ-007 DS-nGMP in the serum samples was determined by MSD. Briefly, the MSD plates were coated with recombinant hPD-1 protein as the capture reagent. Following casein blocking, standards, quality controls, and diluted serum samples (1:200) were added to the plates. Detection was performed with a blocking anti-UDIZ-007 idiotype antibody obtained by rabbit immunization at Genscript (Piscataway, NJ, USA) and conjugated to a Sulfo-Tag. The ECL signal was measured using an MSD Sector Imager reader. The assay quantification range was 1000 to 50,000 ng/mL. Pharmacokinetic parameters, including C_0_, Cmax, AUC_0−1008h_ᵣ, (t_1_/_2_), clearance (Cl), and volume of distribution (Vz), were calculated from individual concentration–time profiles using non-compartmental analysis (NCA) with Phoenix WinNonlin^®^ software (version 8.3).

### 4.7. Repeated-Dose Toxicity in Cynomolgus Macaques

The repeated-dose toxicity study with a recovery group was conducted in cynomolgus macaques approximately 29–44 months of age (n = 3/sex/group for the main study; n = 1/sex/group for recovery period). Animals received seven weekly IV doses on days 1, 8, 15, 22, 29, 35, and 42, administered as a slow bolus. Treatments consisted of a vehicle control (formulation buffer) and three UDIZ-007 DS-nGMP doses (10, 50, and 101.2 mg/kg). The main study phase was completed on day 43, while a subset of animals was maintained for an additional 28-day treatment-free recovery period and evaluated on day 71 to assess the reversibility, persistence, or delayed onset of treatment-related findings.

Daily clinical observations and weekly detailed evaluations were performed during the study, along with body weight and food consumption measurements. Ophthalmologic examinations (pre-treatment and prior to necropsy) and electrocardiographic (ECG) assessments (pre-dose and 1–2 h post-dose on day 42) were conducted to evaluate potential cardiovascular effects.

Blood samples were analyzed for hematology (red and white blood cell counts, hemoglobin, hematocrit), coagulation factors (prothrombin time, activated partial thromboplastin time, and fibrinogen), and clinical chemistry parameters (liver enzymes, renal function, electrolytes, glucose, and lipids). Urinalysis included evaluation of urine volume, color, pH, protein, glucose, ketones, and blood.

For toxicokinetic (TK) evaluation, blood samples were collected at pre-dose and at 0.083 h post-dose and on days 1, 8, 15, 22, 29, 35, and 42. Quantification of UDIZ-007 DS-nGMP in serum was performed using the methodology described above for the single-dose pharmacokinetic study. TK parameters, including trough concentrations (Ctrough), were evaluated to characterize systemic exposure following repeated administration.

On the scheduled termination day (day 43 for the main study and day 71 for recovery animals), complete necropsy examinations were performed, and tissues were collected for organ weight determination and histopathological evaluation.

### 4.8. Euthanasia and Necropsy

Main study and recovery animals surviving to scheduled termination were weighed and euthanized by intravenous administration of a euthanasia solution under sedation, followed by exsanguination. When possible, animals were euthanized in a rotating sequence across dose groups, including controls, to minimize potential time-of-day effects on necropsy evaluations.

Complete necropsy examinations were performed on all animals and included evaluation of the carcass and musculoskeletal system, external surfaces and orifices, cranial cavity and external surfaces of the brain, and thoracic, abdominal, and pelvic cavities with their associated organs and tissues. Selected organs (brain, heart, liver, kidneys, spleen, and thymus) were weighed, and tissues from a comprehensive panel, including lymphoid organs, gastrointestinal tissues, central nervous system tissues, and injection sites, were collected. Tissues were fixed in 10% neutral-buffered formalin (except eyes and testicles, which were fixed in specialized solutions), processed, stained with hematoxylin and eosin (H&E), and examined microscopically by a certified veterinary pathologist. Necropsy procedures were conducted by qualified personnel trained in animal anatomy and gross pathology, with veterinary pathology oversight throughout the procedure.

### 4.9. Anti-Drug Antibodies

The immunogenic response against UDIZ-007 DS-nGMP was assessed by measuring ADA levels in the serum samples of the single-dose PK study and the serum samples from day 42 post-treatment and day 71 (end of the recovery period). ADA detection was performed by MSD, validated according to FDA and EMA guidelines [27,30]. Briefly, serum samples were diluted 1:100 (minimum required dilution) and incubated with a mixture of biotin-conjugated UDIZ-007 DS-nGMP and Sulfo-Tag-conjugated UDIZ-007 DS-nGMP. The formed immune complexes were captured on a streptavidin-coated plate via the biotin-conjugated UDIZ-007 DS-nGMP and revealed with the Sulfo-Tag-conjugated UDIZ-007 DS-nGMP. The ECL signal was measured using an MSD reader. The cutoff point factor for declaring a sample as ADA-positive was established at 1.20 Signal/Noise (S:N) ratio. The results obtained from the method validation are summarized in the Supplementary Material, Table S4.

### 4.10. Tissue Cross-Reactivity in Human and Cynomolgus Macaque Tissues

The specificity of UDIZ-007 DS-nGMP was evaluated at HistologiX (Nottingham, UK) under GLP conditions. Analyses were performed in a comprehensive panel of 32 human and cynomolgus macaque tissues. The tissues included adrenal gland, bone marrow, blood (human only), brain (cerebellum and cerebrum), breast, eye, fallopian tube, stomach, small and large intestine, heart, kidney, liver, lung, lymph node, ovary, pancreas, parathyroid gland, peripheral nerve, pituitary gland, prostate, parotid salivary gland, skin, spinal cord, spleen, striated muscle, testis, thymus, thyroid, tonsil, ureter, urinary bladder, uterus (cervix and endometrium), and placenta (human only). The samples were obtained from three different donors per tissue from commercial and ethical tissue banks. Fresh frozen tissues were cryosectioned at 5–8 μm thickness, mounted on SuperFrost Plus slides, and air-dried. Tissue integrity was confirmed by immunohistochemical (IHC) staining with an anti-vimentin antibody (Abcam, rabbit recombinant; Cat. No. ab92547, Cambridge, UK).

For the final IHC assay, tissue sections were fixed with 4% paraformaldehyde and incubated with UDIZ-007 DS-nGMP at three concentrations (optimal: 1.25 μg/mL, supra-optimal: 2.5 μg/mL, and sub-optimal: 0.625 μg/mL), an isotype control (D92C), and a buffer negative control. Control tissues were included in each run (human tonsil and human cerebellum as positive and negative controls, respectively). Detection was performed using an anti-FITC secondary antibody (Bio-Rad, rabbit, Cat. No. 4510-7804, Kidlington, UK) at 2.0 μg/mL, followed by an anti-rabbit HRP-conjugated polymer (Abcam, goat, code ab214880, Cambridge, UK) and developed with DAB chromogen (Vector Labs, ImmPACT DAB, Cat. No. SK-4105, Newark, CA, USA). Normal goat serum (Vector Labs, code S-1000-20, Newark, CA, USA) and 10% casein (Vector Labs, Cat. No. SP-5020, Newark, CA, USA) were used for blocking nonspecific binding.

Staining evaluation was performed using light microscopy by a certified pathologist, who assigned semi-quantitative scores for intensity (0 = negative, 1 = minimal, 2 = mild, 3 = moderate, 4 = marked) and staining distribution (−=no cells, +=very rare cells, ++=rare cells, +++=occasional cells, ++++=frequent cells), recording subcellular localization (cytoplasmic/membranous) and the specific cell type showing positive binding.

## 5. Conclusions

The fully human anti-PD-1 antibody UDIZ-007 demonstrated robust and durable anti-tumor activity, predictable pharmacokinetic behavior, selective target-associated tissue binding, and good tolerability in relevant preclinical models. Repeated administration in cynomolgus macaques identified no major treatment-related toxicities and established a NOAEL of 101.2 mg/kg. Together, these results provide a comprehensive non-clinical data package supporting the advancement of UDIZ-007 into clinical evaluation as a novel cancer immunotherapy.

## 6. Patents

Patent applications covering the anti-PD-1 antibody UDIZ-007 and related inventions described in this study including the U.S. Patent Application No. 19/396,723 and PCT Application No. WO 2026/112407 A1 have been filed.

## Figures and Tables

**Table 1:** ka, kd and K_D_ values determined by SPR using IgG capture and by BLI using HIS1K biosensors.

	ka (M^−1^ s^−1^)		kd (s^−1^)		K_D_ (nM)	
Method	UDIZ-007-DS	Pembrolizumab	UDIZ-007-DS	Pembrolizumab	UDIZ-007-DS	Pembrolizumab
SPR—IgG Capture	7.42 × 10^5^	1.51 × 10^6^	1.83 × 10^−3^	3.15 × 10^−3^	2.47	2.09
BLI—HIS1K Capture *	4.14 × 10^5^	5.67 × 10^5^	<1.00 × 10^−6^	<1.00 × 10^−6^	<0.0024	<0.0018

(*) The dissociation rates (kd) were below the BLI detection limit (1.0 × 10^−6^ s^−1^). Thus, K_D_ values were calculated using 1.0 × 10^−6^ s^−1^.

**Table 2:** Summary of efficacy results in the B-hPD-1 mouse model.

G	Treatment	n (Day28)	TV (mm^3^) ^a^ (Day28)	% TGI ^b^ (Day28)	% TR ^c^ (Day28)	CR ^d^ (Day 56)	Median Survival (Day) ^e^	Max %BWL (Day) ^f^
**1**	D92C 10 mg/kg	10	964.61 ± 235.33	-	-	2/10 (20%)	38.5	2.02 (Day 17)
**2**	Pembrolizumab 10 mg/kg	10	11.51 ± 11.51 ***	100%	88.49%	9/10 (90%)	Undefined ***	4.93 (Day 3)
**3**	UDIZ-007 10 mg/kg	10	0.00 ± 0.00 ****	100%	100%	10/10 (100%)	Undefined ***	4.99 (Day 3)
**4**	UDIZ-007 2.5 mg/kg	10	35.77 ± 35.77 ***	100%	64.23%	9/10 (90%)	Undefined ***	5.93 (Day 3)
**5**	UDIZ-007 1 mg/kg	10	192.27 ± 115.07 *	89.33%	-	7/10 (70%)	Undefined **	3.73 (Day 3)
**6**	DPBS	10	936.29 ± 168.74	3.27%	-	0/10 (0%)	36.5	12.30 (Day 17)

**a** Mean ± SEM of TV on Day 28, the last time point at which all animals remained on study (n = 10 per group). Statistical comparisons were performed versus the G1 D92C isotype control group using the Kruskal–Wallis test followed by Dunn’s multiple comparisons test. **b** TGI (%) = [1 − (Tt − T0)/(Ct − C0)] × 100%, where Tt = mean TV of treated at time t, T0 = mean TV of treated at time 0 (baseline), Ct = mean TV of control at time t and C0 = mean TV of control at time 0 (baseline). **c** TR (%) = [1 − (Tt/T0)] × 100%. **d** CR = Complete Response or Complete Tumor Regression. No tumor on Day 56. **e** Days following the start of treatment. Day 0 = day of treatment initiation. Compared to G1_ D92C control group by Log Rank (Mantel–Cox) test. **f** The highest individual % BWL value observed in that group and the time point observed. * 0.01 < *p* < 0.05, ** 0.001 < *p* < 0.01, *** 0.0001 < *p* < 0.001, **** *p* < 0.0001. TV: tumor volume; TGI: Tumor growth inhibition; TR: Tumor Regression; CR: Complete Response; BWL: Body Weight Loss.

**Table 3:** Summary of repeated-dose toxicology findings following IV administration of UDIZ-007 DS-nGMP in cynomolgus macaques.

Parameter	Result
**Mortality**	None
**Clinical observations**	No treatment-related findings
**Body weight**	No treatment-related effects
**Food consumption**	No treatment-related findings
**Ophthalmology**	No treatment-related findings
**Electrocardiography**	No treatment-related abnormalities
**Hematology**	No treatment-related effects
**Coagulation**	No treatment-related effects
**Clinical chemistry**	No treatment-related effects
**Urinalysis**	No treatment-related effects
**Organ weights**	No treatment-related changes
**Macroscopic pathology**	No treatment-related findings
**Microscopic pathology**	Mild to moderate reversible mononuclear cell infiltrates in choroid plexus at 50 and 101.2 mg/kg/dose
**Recovery assessment**	No treatment related microscopic findings after the 28-day recovery period
**PK/TK profile**	Sustained systemic exposure throughout repeated dosing
**ADA**	Detected in subset of animals; associated with reduced circulating drug concentrations
**NOAEL**	101.2 mg/kg/dose

## Data Availability

The original contributions presented in this study are included in the article/Supplementary Material. Further inquiries can be directed to the corresponding authors.

## Supplementary Materials

The following supporting information can be downloaded at https://www.mdpi.com/article/10.3390/ph19091423/s1, Figure S1: Biophysical characterization of UDIZ-007 DS-nGMP; Figure S2. Representative SPR and BLI sensorgrams of UDIZ-007 DS-nGMP and pembrolizumab binding to human PD-1 (hPD-1); Table S1: Anti-Drug Antibodies (ADAs) in cynomolgus macaques following single intravenous administration of UDIZ-007 DS-nGMP; Table S2: Anti-Drug Antibodies (ADAs) in cynomolgus macaques following repeated intravenous administration of UDIZ-007 DS-nGMP; Table S3: Tissue cross-reactivity of UDIZ-007 DS-nGMP in human and cynomolgus macaque tissues; Table S4. Summary of results obtained during the validation of the screening method for detecting ADAs in monkey serum samples.

## Abbreviations

The following abbreviations are used in this manuscript:

ADA	Anti-Drug Antibody
AUC	Area Under the Concentration–Time Curve
AUC_0-t_	Area Under the Concentration–Time Curve from Time 0 to Last Measured Time Point
AUC_0-∞_	Area Under the Concentration–Time Curve Extrapolated to Infinity
AUC_0-1008hr_	Area Under the Concentration–Time Curve from 0 to 1008 Hours
B-hPD-1	Human PD-1 Transgenic Mouse Model
BLI	Biolayer Interferometry
BSA	Bovine Serum Albumin
BW	Body Weight
BWL	Body Weight Loss
CHO	Chinese Hamster Ovary
Cl	Clearance
Cmax	Maximum Serum Concentration
Cmin	Minimum Serum Concentration
CR	Complete Response
Ct	Control Group Tumor Volume at Time t
Ctrough	Trough Concentration
C0	Control Group Baseline Tumor Volume
DAB	3,3′-Diaminobenzidine
DPBS	Dulbecco’s Phosphate Buffered Saline
DS-nGMP	Drug Substance, Non-Good Manufacturing Practice
DTT	Dithiothreitol
EC50	Half-Maximal Effective Concentration
ECG	Electrocardiogram
ECL	Electrochemiluminescence
EMA	European Medicines Agency
FDA	United States Food and Drug Administration
GLP	Good Laboratory Practice
GMP	Good Manufacturing Practice
H&E	Hematoxylin and Eosin
HED	Human Equivalent Dose
hPD-1	Human Programmed Cell Death Protein 1
hPD-L1	Human Programmed Death Ligand 1
HRP	Horseradish Peroxidase
IACUC	Institutional Animal Care and Use Committee
IHC	Immunohistochemistry
IgG	Immunoglobulin G
IGHV	Immunoglobulin Heavy Variable Gene
IP	Intraperitoneal
IV	Intravenous
ka	Association Rate Constant
kd	Dissociation Rate Constant
K_D_	Equilibrium Dissociation Constant
kDa	Kilodalton
LALA	L234A/L235A Fc Mutations
MOA	Mechanism of Action
MS	Mass Spectrometry
MSD	Meso Scale Discovery
NCA	Non-Compartmental Analysis
NFAT	Nuclear Factor of Activated T Cells
NOAEL	No-Observed-Adverse-Effect Level
PBS	Phosphate Buffered Saline
PD-1	Programmed Cell Death Protein 1
PD-L1	Programmed Death Ligand 1
PD-L2	Programmed Death Ligand 2
PK	Pharmacokinetics
QCs	Quality Controls
SDS-PAGE	Sodium Dodecyl Sulfate Polyacrylamide Gel Electrophoresis
SEC-UPLC	Size-Exclusion Chromatography Ultra-Performance Liquid Chromatography
SEM	Standard Error of the Mean
SPR	Surface Plasmon Resonance
S:N	Signal-to-Noise Ratio
TCR	Tissue Cross-Reactivity
TGI	Tumor Growth Inhibition
TK	Toxicokinetics
TR	Tumor Regression
TV	Tumor Volume
t½	Terminal Elimination Half-Life
Tmax	Time to Maximum Concentration
UDIZ-007 DS-nGMP	UDIZ-007 Drug Substance Non-GMP Preparation
UPLC	Ultra-Performance Liquid Chromatography
Vd	Volume of Distribution
Vz	Volume of Distribution During Terminal Phase
