# Supplementary material for: Non-Clinical Evaluation of the Anti-PD-1 Antibody UDIZ-007
Source: Pharmaceuticals (Basel). 2026 Sep 9;19(9):1423. doi: 10.3390/ph19091423 (PMC13610128; doi:10.3390/ph19091423)
Supplement: Supplementary file 1 [file pharmaceuticals-19-01423-s001.zip › pharmaceuticals-4435835-supplementary.pdf]

Supplementary Material

Non-Clinical Evaluation of the Anti-PD-1 Antibody UDIZ-007

Gregorio de Jesús Carballo Uicab [1,2], Keyla María Gómez Castellano [1,2], Frida Daniela Ramírez Villedas [1,2], Marco A. Velasco Velázquez [3], Ileana Licona-Limón [1,2], Said Kayum Vázquez Leyva [1,2], Hugo Alberto Barrera Saldaña [2,4], Sonia Mayra Pérez Tapia [1,2,5,*] and Juan Carlos Almagro [1,6,*]

Figure S1. Highlights of the biophysical characterization of UDIZ-007 DS-nGMP.

Figure S2. Representative SPR and BLI sensorgrams of UDIZ-007 DS-nGMP and pembrolizumab binding to human PD-1 (hPD-1).

Table S1. Anti-Drug Antibodies (ADAs) in cynomolgus macaque at single IV doses of 10, 50, and 101.2 mg/kg.

Table S2. Anti-Drug Antibodies (ADAs) in cynomolgus macaque assessed on day 42 and 71 in the repeated IV administrations.

Table S3. Cross-reactivity in human and cynomolgus macaque tissues.

Table S4. Summary of results obtained during the validation of the screening method for detecting ADAs in monkey serum samples.

Figure S1. Biophysical characterization of UDIZ-007 DS-nGMP. (A) SDS-PAGE analysis of UDIZ-007 DS-nGMP under non-reducing (NR) and reducing (R) conditions. Under non-reducing conditions, a predominant band corresponding to intact IgG was observed, whereas reducing conditions revealed the expected heavy and light chains. Molecular weight marker (MW) positions are indicated. (B) SEC-UPLC chromatogram showing a predominant monomeric species with no detectable high-molecular-weight aggregates. (C) Deconvoluted intact mass spectrum of UDIZ-007 DS-nGMP showing the distribution of the major glycoforms. The predominant species corresponded to the G0F/G0F glycoform (146,855 Da), together with minor G0/G1F (146,648 Da), G0F/G1F (147,019 Da), and G1F/G1F (147,182 Da) glycoforms.

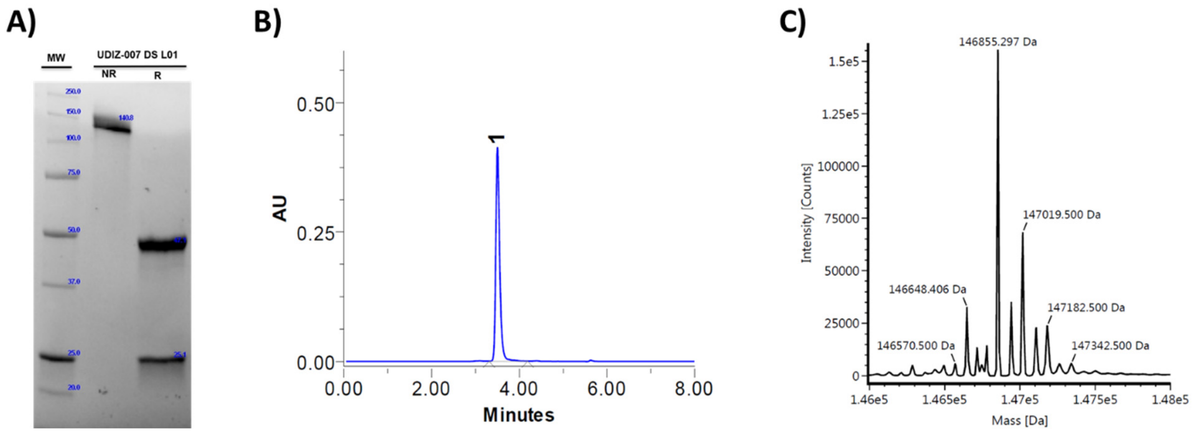

Figure S2. Representative SPR and BLI sensorgrams of UDIZ-007 DS-nGMP and pembrolizumab binding to human PD-1 (hPD-1). For the SPR measurements, two different capture formats were used: anti-human IgG and Protein A. The anti-human IgG and Protein A were covalently attached to CM5 sensor chips. UDIZ-007 and pembrolizumab were captured by the anti-human IgG or Protein A sensor chips, and recombinant hPD-1 (6.25–50 nM) was injected as the analyte. Association and dissociation were monitored for 150 and 300 s, respectively, and the sensorgrams were fitted using a 1:1 Langmuir binding model. For BLI analysis, recombinant PD-1-His (5 µg/mL) was immobilized on HIS1K biosensors, and binding of serial dilutions of each antibody (1.56–100 nM) was evaluated with association and dissociation phases of 90 s each. Pembrolizumab was included as the reference antibody and processed side-by-side with UDIZ-007 DS-nGMP.

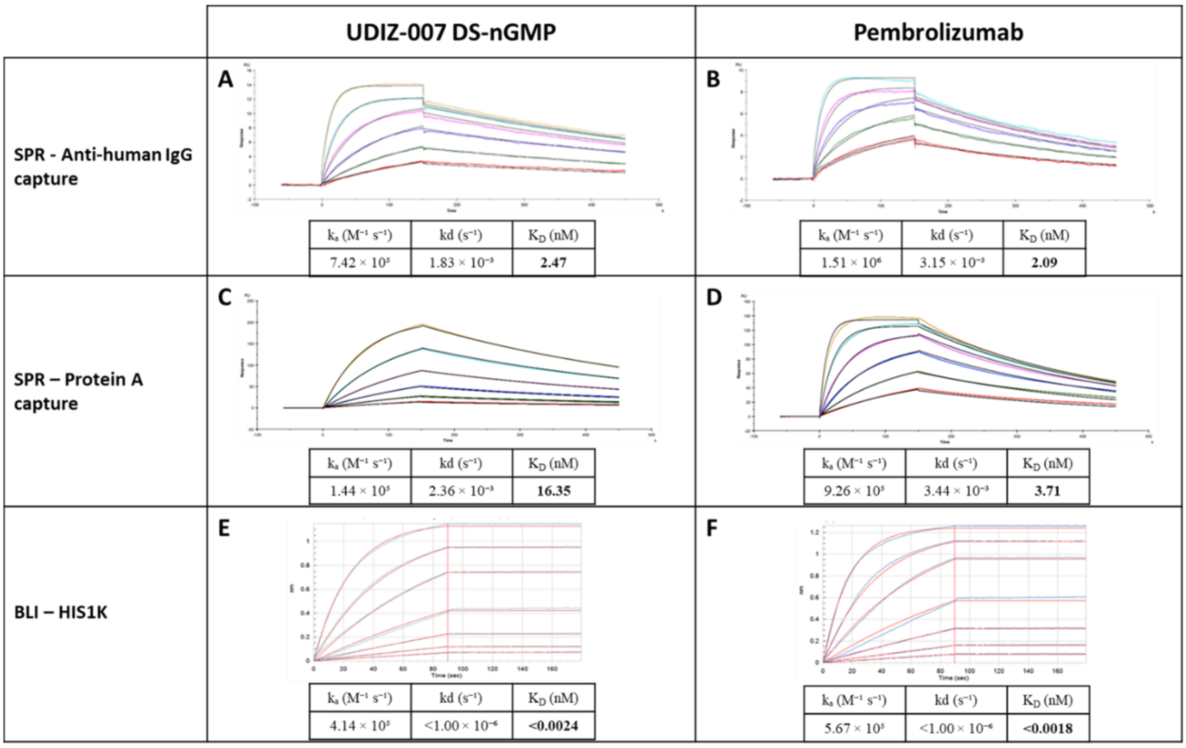

Table S1. Anti-Drug Antibodies (ADAs) in cynomolgus monkey at single IV doses of 10, 50, and 101.2 mg/kg.

**Negative.**
**Value above the SCPF but close to the cut-off point (1.2).**
**Clearly positive value.**

Sampling time (h)	10 mg/kg		50 mg/kg		101.2 mg/kg	
	Male (1001)	Female (1501)	Male (2001)	Female (2501)	Male (3001)	Female (3501)
**0**	0.931	0.937	0.910	0.931	0.966	0.971
**0.083**	1.010	0.963	0.921	0.921	0.981	0.893
**1**	0.979	0.931	0.889	0.942	0.961	0.913
**6**	1.020	0.963	0.958	0.898	0.888	0.927
**24**	1.040	0.937	0.989	0.917	0.898	0.893
**48**	0.894	0.899	1.000	0.947	0.908	0.883
**72**	0.910	0.899	0.952	0.942	0.908	0.947
**168**	0.910	0.931	0.979	0.932	0.942	0.869
**336**	4.340	1.120	1.000	0.947	0.947	0.908
**504**	165	1.350	1.040	0.951	0.951	0.869
**672**	123	1.430	1.010	0.971	0.937	0.951
**840**	139	1.400	0.873	1.040	0.927	0.966
**1008**	188	1.390	0.868	1.130	0.917	0.932

Individual ADA assay results (S:N ratio) for each cynomolgus monkey throughout the study.
S:N, signal-to-noise ratio.

Table S2. Anti-Drug Antibodies (ADAs) in cynomolgus monkey assessed on day 42 and 71 in the repeated IV administrations.

**Negative.**
**Value above the SCPF but close to the cut-off point (1.2).**
**Clearly positive value.**

Dose (mg/kg)	Number & Gender (M/F)	Sampling time (day nominal)	S:N
**0**	**4001-M**	42	2.61
	**4001-M**	71	2.52
	**4002-M**	42	1.05
	**4501-F**	42	1.41
	**4501-F**	71	1.02
	**4502-F**	42	1.06
**10**	**5001-M**	42	1.06
	**5002-M**	42	1.23
	**5003-M**	42	3,700
	**5501-F**	42	1.92
	**5502-F**	42	1.42
	**5503-F**	42	642
**50**	**6001-M**	42	1.06
	**6001-M**	71	0.94
	**6002-M**	42	1.08
	**6003-M**	42	2.09
	**6004-M**	42	1.05
	**6501-F**	42	1.18
	**6501-F**	71	0.97
	**6502-F**	42	1.58
	**6503-F**	42	92.40
	**6504-F**	42	1.22
**101.2**	**7001-M**	42	3.39
	**7001-M**	71	1.51
	**7002-M**	42	0.93
	**7003-M**	42	0.96
	**7004-M**	42	3.91
	**7501-F**	42	1.27
	**7501-F**	71	1.03
	**7502-F**	42	1.97
	**7503-F**	42	1.14
	**7504-F**	42	1.01

Table S3. Cross-reactivity in human and cynomolgus macaque tissues. Semi-quantitative scores for intensity (0=negative, 1=minimal, 2=mild, 3=moderate, 4=marked) and staining distribution (−=no cells, +=very rare cells, ++=rare cells, +++=occasional cells, ++++=frequent cells), recording subcellular localization (cytoplasmic/membranous) and the specific cell type showing positive binding.

Tissue/Donor	Human				Cynomolgus macaque			
	Finding	Positive tissues/Total tissues	Intensity	Distribution	Finding	Positive tissues/Total tissues	Intensity	Distribution
Adrenal Gland	No specific positive staining	3/3	0	-	No specific positive staining	3/3	0	-
Blood Cells	No specific positive staining	3/3	0	-	Not evaluated	NA	NA	NA
Bone Marrow	No specific positive staining	3/3	0	-	No specific positive staining	3/3	0	-
Brain (Cerebellum)	No specific positive staining	3/3	0	-	No specific positive staining	3/3	0	-
Brain (Cerebrum)	No specific positive staining	3/3	0	-	No specific positive staining	3/3	0	-
Breast	No specific positive staining	3/3	0	-	No specific positive staining	3/3	0	-
Eye	No specific positive staining	3/3	0	-	No specific positive staining	3/3	0	-
Fallopian Tube	No specific positive staining	3/3	0	-	No specific positive staining	3/3	0	-
Stomach	Lymphocytes; within the gut-associated lymphoid tissue	2/3	2	+	Lymphocytes; within the gut-associated lymphoid tissue	2/3	3	+/++
	No specific positive staining	1/3	0	-	No specific positive staining	1/3	0	-
Small Intestine	Lymphocytes; within the gut-associated lymphoid tissue	2/3	2	+	Lymphocytes; within the gut-associated lymphoid tissue	3/3	3 - 4	+/++
	No specific positive staining	1/3	0	-				
Large Intestine	No specific positive staining	2/3	0	-				
	Lymphocytes; within the gut-associated lymphoid tissue	1/3	2	+	Lymphocytes; within the gut-associated lymphoid tissue	3/3	2 - 3	+
Heart	No specific positive staining	3/3	0	-	No specific positive staining	3/3	0	-
Kidney	No specific positive staining	3/3	0	-	No specific positive staining	3/3	0	-
Liver	No specific positive staining	2/3	0	-	No specific positive staining	3/3	0	-
	Lymphocytes; within scattered cells	1/3	2	+				
Lung	No specific positive staining	3/3	0	-	No specific positive staining	1/3	0	-

Tissue/Donor	Human				Cynomolgus macaque			
	Finding	Positive tissues/Total tissues	Intensity	Distribution	Finding	Positive tissues/Total tissues	Intensity	Distribution
					Lymphocytes; within scattered cells	2/3	3	+
Lymph Node	Lymphocytes; within cortical germinal centres and scattered cells	3/3	3	+	Lymphocytes; within cortical germinal centres and scattered cells	3/3	4	++/+++
Ovary	No specific positive staining	3/3	0	-	No specific positive staining	3/3	0	-
Pancreas	No specific positive staining	3/3	0	-	No specific positive staining	3/3	0	-
Parathyroid Gland	No specific positive staining	3/3	0	-	No specific positive staining	3/3	0	-
Peripheral Nerve	No specific positive staining	3/3	0	-	No specific positive staining	3/3	0	-
Parotid Salivary Gland	No specific positive staining	3/3	0	-	No specific positive staining	3/3	0	-
Pituitary Gland	No specific positive staining	3/3	0	-	No specific positive staining	3/3	0	-
Placenta	No specific positive staining	3/3	0	-	No specific positive staining	3/3	0	-
Prostate Gland	No specific positive staining	3/3	0	-	No specific positive staining	3/3	0	-
Skin	No specific positive staining	3/3	0	-	No specific positive staining	3/3	0	-
Spinal Cord	No specific positive staining	3/3	0	-	No specific positive staining	3/3	0	-
Spleen	Lymphocytes; within scattered cells	3/3	2 - 3	+	Lymphocytes; within germinal centres and scattered cells	3/3	3 - 4	++/+++
Striated Muscle	No specific positive staining	3/3	0	-	No specific positive staining	3/3	0	-
Testis	No specific positive staining	1/3	0	-	No specific positive staining	3/3	0	-
	Lymphocytes; within lymphoid aggregates and scattered cells	2/3	1 - 3	+/++				
Thymus	Lymphocytes; within medullary regions and scattered cells	3/3	1 - 2	+/++	Lymphocytes; within scattered	3/3	2 - 3	+
Thyroid Gland	No specific positive staining	2/3	0	-	No specific positive staining	3/3	0	-
	Lymphocytes; within a lymphoid aggregate and scattered cells	1/3	2	+				
Tonsil	Lymphocytes; within cortical germinal centres and scattered cells	3/3	3 - 4	++/+++	Lymphocytes; within cortical germinal centres and scattered cells	3/3	3	+++

Tissue/Donor	Human				Cynomolgus macaque			
	Finding	Positive tissues/Total tissues	Intensity	Distribution	Finding	Positive tissues/Total tissues	Intensity	Distribution
Ureter	No specific positive staining	3/3	0	-	No specific positive staining	3/3	0	-
Urinary Bladder	Lymphocytes; within lymphoid aggregates and scattered cells	3/3	1 - 3	+/++	No specific positive staining	3/3	0	-
Uterus (Cervix)	No specific positive staining	3/3	0	-	No specific positive staining	3/3	0	-
Uterus (Endometrium)	No specific positive staining	3/3	0	-	No specific positive staining	2/2	0	-

Gray-shaded cells indicate that no additional immunohistochemical finding was observed for the corresponding species.

Table S4. Summary of results obtained during the validation of the screening method for detecting ADAs in monkey serum samples.

Parameter	Result
Screening Cut Point	Fifteen individual matrix lots were analyzed in duplicate wells within 4 runs utilizing 2 analysts.<br>A total of 60 data points were used to determine the cut point. Three data points were identified as analytical outliers (batches 13–15, series 3) and two subjects as biological outliers (batch 3 and batch 6), resulting in the exclusion of 11 data points from the cut point analysis. A parametric approach was selected to determine the screening cut point using the 99th percentile of the 49 valid data points, yielding a screening cut point value of 1.20.
Sensitivity	Sensitivity results for the screening assay were as follows: Run 1: 3.91 ng/mL; Run 2: 1.95 ng/mL; Run 3: 1.95 ng/mL; Run 4: 3.91 ng/mL. All results were within ±1 2-fold dilution difference which is expected due to daily standard assay fluctuations. The lowest concentration tested that was consistently ≥ plate specific cut point for all curves was 3.91 ng/mL, which was considered to be the screening limit of sensitivity for this procedure.
Precision	Precision was assessed by intra- and inter-assay variability using Negative Control (NC; naïve pooled serum), Low Positive Control (LPC; 100 ng/mL of anti-UDIZ-007), and High Positive Control (HPC; 1000 ng/mL of anti-UDIZ-007). Intra-assay %CVs were 2.9% (NC), 5.9% (LPC), and 3.9% (HPC). Inter-assay %CVs were 8.4% (NC), 18.1% (LPC), and 15.6% (HPC). All precision results met the established acceptance criteria.
Drug Tolerance	Positive control at 100 ng/mL = 750 μg/mL<br>Positive control at 1000 ng/mL= 1250 μg/mL
Short-Term Stability	18 hours at ambient temperatures
Freeze-Thaw Cycle Stability	3 cycles in a freezer set to -60°C to -90°C, thawed at ambient temperatures
Selectivity	All of the individual lots generated signal responses below the screening cut point when unfortified and above the screening cut point when fortified at low positive control.<br>The hemolyzed lot generated a signal response below the screening cut point when unfortified and above the screening cut point when fortified at low positive control.
Conclusion	A qualitative, screen-only immunoassay for the detection of anti-UDIZ-007 antibodies in non-human primate serum was successfully validated, with a screening cut point factor of 1.20. The results demonstrated that the validated procedure was sensitive, precise, and selective. Surrogate antibodies to UDIZ-007 were confirmed to be stable during storage and processing.
